# Nanostructured h‑WO_3_‑Based Ionologic Gates with Enhanced Rectification and Transistor Functionality

**DOI:** 10.1021/acsnano.5c02035

**Published:** 2025-05-26

**Authors:** Ahmed Bahrawy, Przemyslaw Galek, Christin Gellrich, Nick Niese, Mohamed A. A. Mohamed, Martin Hantusch, Julia Grothe, Stefan Kaskel

**Affiliations:** † Inorganic Chemistry I, 9169 Technische Universität Dresden, Bergstrasse 66, Dresden 01069, Germany; ‡ 28394 Leibniz Institute for Solid State and Materials Research Dresden, Helmholtzstraße 20, Dresden 01069, Germany; § Department of Physics, Faculty of Science, Sohag University, Sohag 82524, Egypt; ∥ Fraunhofer Institute for Material and Beam Technology (IWS), Winterbergstraße 28, Dresden 01277, Germany

**Keywords:** electrochemical capacitor diode (CAPode), ionic diode, ionic amplifier, ionic transistor, switchable supercapacitor

## Abstract

Iontronic devices link ion-based transport with established electronic systems. Emerging capacitive devices, such as CAPode and G-Cap, feature diode-like rectification and transistor-like switching, respectively, through electrochemical capacitor functionality for enhanced energy storage and signal processing in next-generation low-power electronics. In this study, we present an asymmetric architecture based on nanostructured hexagonal tungsten oxide with significantly enhanced current rectification (with a rectification ratio of 58), providing a performant ionic transistor with 97.5% switching efficiency under only a 1 V bias. Key parameters, such as substrate materials, the mass ratio of the counter electrode to the working electrode, electrolyte composition, and concentration, are evaluated to reach the highest rectification ratios. The final device exhibited remarkable stability, maintaining performance for over 20,000 cycles without degradation. Additionally, integrating a third electrode into the optimized CAPode (termed G-Cap) allowed it to function as a transistor analogue, showing excellent switchability. The third gate electrode in the G-Cap plays a critical role in shifting the working electrode potential to reach the redox potential of tungsten oxide, enhancing the device functionality. As a proof of concept, the CAPodes were integrated into basic and complex logic gates under varying voltages and frequencies up to 1000 mHz, with output signals demonstrating robust performance. In addition, the logic operation metrics revealed a low threshold voltage of 0.4 V and a low power consumption of 2 μW. These results highlight the potential for expanded applications of this device structure.

## Graphical abstract

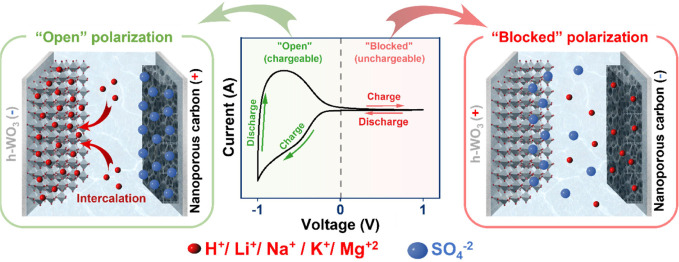

## Introduction

In recent years, iontronic devices have emerged as a ground-breaking field at the intersection of electronics and ionics, promising to bridge the gap between traditional electronic devices and biological systems.^1^ Iontronics leverages the transport and control of ions, akin to how electronics manipulate electrons to interface with complex biological processes driven by ionic signaling.^2^ This novel approach opens new possibilities in neuromodulation, bioelectronics, and energy storage, where precise management of ion flow is essential.^3^ Currently, the ion transport within electrolytes or ionic conductors and their role in processes like electrochemical reactions and biological signaling are studied in ionic systems.^4^ Although ion transport is slower than electron transport, ions are crucial due to their ability to trigger complex chemical reactions and biological responses.^5^ Neurons, synapses, and ion channels play crucial roles in biological logic operations.^6^ While neurons are the primary processing units, synapses and ion channels are essential for transmitting and modulating signals.^6^ Synapses integrate multiple inputs through excitatory and inhibitory signals, effectively performing logic-like operations such as summation, thresholding, and gating. Ion channels regulate the flow of ions, influencing neuronal excitability and signal propagation. In contrast, electronics focus on electron manipulation through conductors (e.g., metals and semiconductors) to process information, generate signals, or power devices thanks to the fast and efficient movement of electrons.^7^ Iontronic systems combine ion transport with electron flow, maintaining the characteristics of electronics, such as logical response, while also leveraging ionic systems for energy storage through chemical reactions.^8^ These devices can control ionic currents to interact with biological tissues, simulate neural functions, and store energy in innovative ways. Unlike electronics, which rely primarily on electrons, iontronics use ions (e.g., Li^+^, Na^+^, K^+^, Mg^2+^), charged atoms or molecules, which play vital roles in biological systems.^9^ The first example of a bioactive ion-based switchable supercapacitor based on choline chloride and porous carbons was introduced recently.^2^ While electronics are crucial for processing information and power delivery, ionics are key for interacting with biological systems, such as neurons communicating through ion flow.^10^ This hybridization facilitates more seamless communication between artificial systems and living organisms, offering unprecedented opportunities in medical devices, neural prosthetics, and brain-machine interfaces in the future.

Electrochemical capacitor (EC) diodes (CAPodes),^8,11^ gated ECs (G-Cap),^3,12^ and gated-assisted switchable EC diodes (G-CAPodes)^13^ represent promising iontronic capacitive devices analogous to electronic diodes, transistors, and variable-output capacitor diodes. The CAPode device, as an asymmetric or hybrid EC, operates across both “positive” and “negative” voltage windows (system repolarization), exhibiting distinct current responses with a diode-like current–voltage (*I*–*U*) characteristic curve. The original CAPode concept was purely based on ion-sieving and capacitive ion storage.^14^ However, recently, hybrid capacitors with at least one faradaic electrode are emerging.^15^ In principle, any accumulator can be regarded as a unidirectional rectification system. In terms of rate and cycling performance, EDLC and hybrid capacitors are highly advantageous. The CAPode devices reported so far are classified according to their working mechanisms based on either ion sieving or asymmetric redox-active mechanisms. The first concept of the CAPode was introduced as an ion-sieving mechanism-dependent device, where rectification behavior was achieved by controlling the pore size distribution in the electrodes to accommodate specific ions of varying sizes.^16^ Subsequently, asymmetric CAPode devices were developed using at least one redox-active material to achieve high current under selected redox-active polarization and low current response under “blocked” polarization.^11c,17^ More recently, redox-active electrolytes have been employed to construct CAPode devices, with their performance determined by the selection of specific electrode materials that undergo redox reactions under “open” polarization while preventing redox reactions under “blocked” polarization. The G-Cap device functions as an ionic transistor, where an internal EC is regulated by a third “gate” electrode, enabling switching between the “on” and “off” state.^3^ The G-CAPode concept introduces modulation of internal capacitance by adjusting the potential of the working electrode (WE) and the counter electrode (CE), with the output being controlled by the “gate” electrode (GE) bias.

The use of ECs in iontronic devices offers significant advantages particularly in terms of energy efficiency, overcoming the limitations of traditional electronics in biological applications. Generally, ECs are ideal for powering iontronic devices due to their ability to store and deliver energy rapidly, a feature essential for controlled ionic transport.^18^ The ability to electrosorb biologically active ions is crucial for applications such as neuromodulation and bioelectronic interfaces. Rapid energy delivery and high efficiency in energy cycling make them suitable for long-term operations with intermittent power demands, such as neural prosthetics and implantable medical devices.^3,19^ In contrast, traditional electronics face limitations when applied to biological systems.^20^ Additionally, electronics generate heat as a byproduct, which can be detrimental to delicate biological structures. ECs mitigate this issue by delivering energy without significant heat generation. To date, the literature on iontronic devices utilizing ECs is limited. Introducing concepts such as CAPode, G-Cap, and G-CAPode devices have advanced research significantly. These devices operate through mechanisms such as ion sieving, battery-like processes, or intercalation. However, the use of organic electrolytes or low rectification restricts their practical applications.

In iontronic applications based on ECs, the intercalation mechanism, ion sieving, and battery-type mechanisms each play a significant role with key differences in their reaction rates.^21^ Intercalation refers to a process where ions (e.g., Li^+^, Na^+^, K^+^, Mg^2^) are inserted into the lattice of a host material without significantly disrupting its structure.^22^ Intercalation can be classified to bulk and surface intercalation.^23,23b^ The surface intercalation mechanism is relatively fast as it allows ions to move quickly in and out of the host material surface, making it suitable for applications requiring rapid ion transport, such as iontronic devices. The reaction rate in surface intercalation is driven by the mobility of ions within the solid structure often enhanced by materials with open frameworks or layered structures, such as tungsten oxide (WO_3_) or other transition metal oxides.^22,24^ In contrast, ion sieving involves selectively allowing certain ions to pass through a membrane or material based on their size or charge. While this method is efficient for filtration or separation purposes, the reaction rate can be slower compared to intercalation, as it is governed by diffusion and the need for ions to navigate through pores or channels.^11b^ Ion sieving is typically used in cases where selectivity is more critical than speed, and its application in iontronics may be limited to processes where controlled ion flux is essential. Finally, battery-type mechanisms involve bulk intercalation and faradaic reactions where ions are involved in redox processes at electrodes. While batteries can store large amounts of energy, their reaction rates are slower than those of surface intercalation mechanisms because ion transport is often coupled with electrochemical reactions which can introduce kinetic limitations.^25^ This makes battery-type with bulk intercalation systems less ideal for real-time iontronic applications where fast switching and response times are required. Therefore, the surface intercalation mechanism offers a balance of speed and efficiency, making it the most suitable for iontronic devices that rely on rapid ion movement for signal modulation or energy transfer. Recently, a complementary metal oxide semiconductor (CMOS)-based supercapacitor diode based on a solid electrolyte has been reported. The CAPode device achieves rectification capability by tuning the redox peaks. Owing to its all-solid-state configuration, this CAPode demonstrates valuable potential for miniaturization and high process integration compatibility.^17^

In this study, we propose a new CAPode based on nanostructured hexagonal tungsten oxide (h-WO_3_) and G-Cap architecture employing biocompatible electrolytes, including Li_2_SO_4_, Na_2_SO_4_, K_2_SO_4_, MgSO_4_, and H_2_SO_4_ (the latter is used for the fundamental studies).^26^ Electrochemical techniques such as cyclic voltammetry (CV), galvanostatic charge–discharge (GCD), and electrochemical impedance spectroscopy (EIS) are used to evaluate and optimize the device performance. Specifically, we investigated the effects of mass loading ratio, electrolyte concentration, and electrolytes on the device response. The proposed devices demonstrate much higher rectification ratios compared to those reported in the literature, exhibiting excellent logic operation for simple AND and OR gates and more complex logic gates by using six devices for logic operation. Furthermore, the switchability of the proposed device is tested and demonstrates appropriate functionality for iontronic applications.

## Results and Discussion

The device architecture features a nanosized oxide-electrode (h-WO_3_@Ti) as WE and nanoporous carbon (NC) as CE. An extra GE (also made from NC) is used for the G-Cap device to control the capacity. The operating mechanism of CAPode is schematically represented (Figure 1).

**Figure 1:**
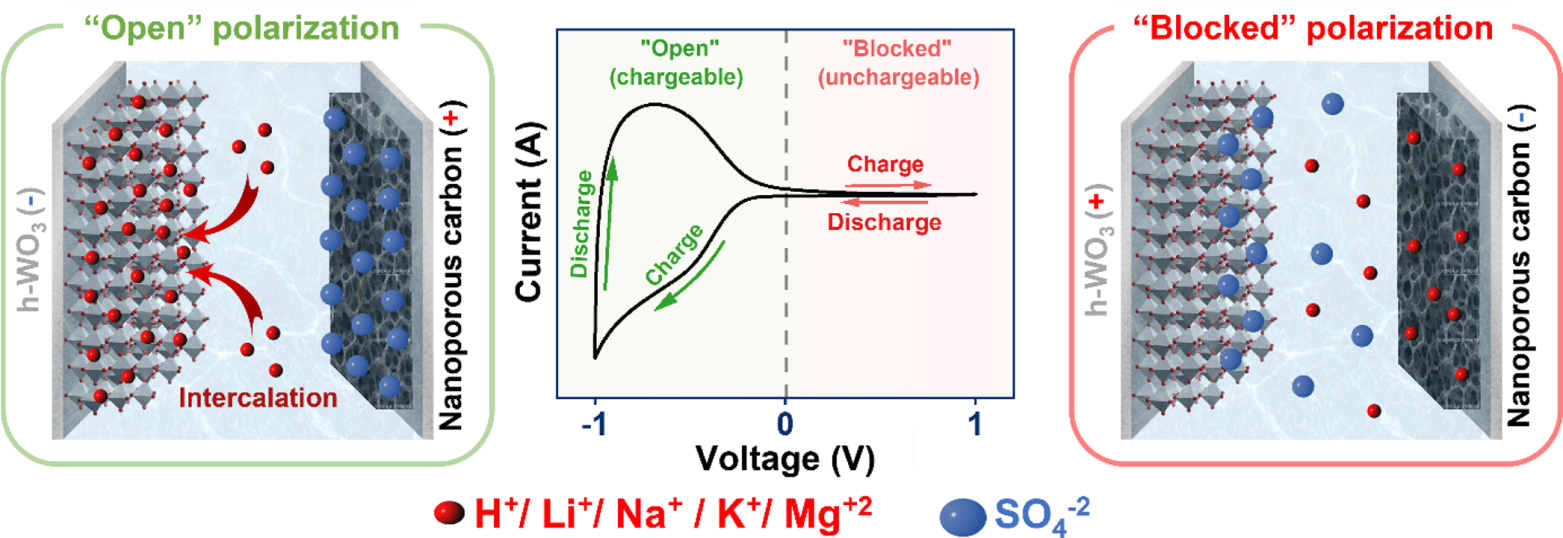
Mechanism of CAPode (h-WO_3_@Ti|0.5 mol L^–1^ H_2_SO_4_|NC@Ti) performance.

The working mechanism involves symmetric polarization of the device and the output current, which is characterized by two distinct current responses. Under “positive” voltage, the current response is minimum, and the polarization is noted as “blocked/unchargeable” polarization. In the other direction, a high current response is registered, and the polarization is noted as “open/chargeable” polarization (*U* < 0 V). The unidirectional current flow of the CAPode device is achieved by controlling the device reaction under “open” polarization and eliminating the reaction under “blocked” polarization. Under “open” polarization, a high current response originated from cations (e.g., H^+^, Li^+^, Na^+^, K^+^, Mg^2^^+^) intercalated to the nanosized h-WO_3_, and the charge balance is achieved by adsorption of counterions to NC. In contrast, under “blocked” polarization, the anions are not able to intercalate to nanosized h-WO_3_ and the current is minimized. The G-Cap device operates with a potential shift concept to control the output capacity and current. The GE is utilized to finely control the reaction rate occurring in the WE (intercalation). Different biases are tested and optimized to reach the transistor-like performance.

### Synthesis and Characterization of Nanostructured h-WO_3_ Electrode Materials

In order to synthesize a highly competitive nanosized oxide-electrode, h-WO_3_ was synthesized via hydrothermal precipitation as powder or directly deposited under identical conditions on titanium or nickel substrates (Figure S1). The chemical composition, oxidation states, and crystallinity of the prepared h-WO_3_ sample were investigated directly on the Ti substrate by using X-ray photoelectron spectroscopy (XPS) and X-ray powder diffraction (XRD; Figure 2).

**Figure 2:**
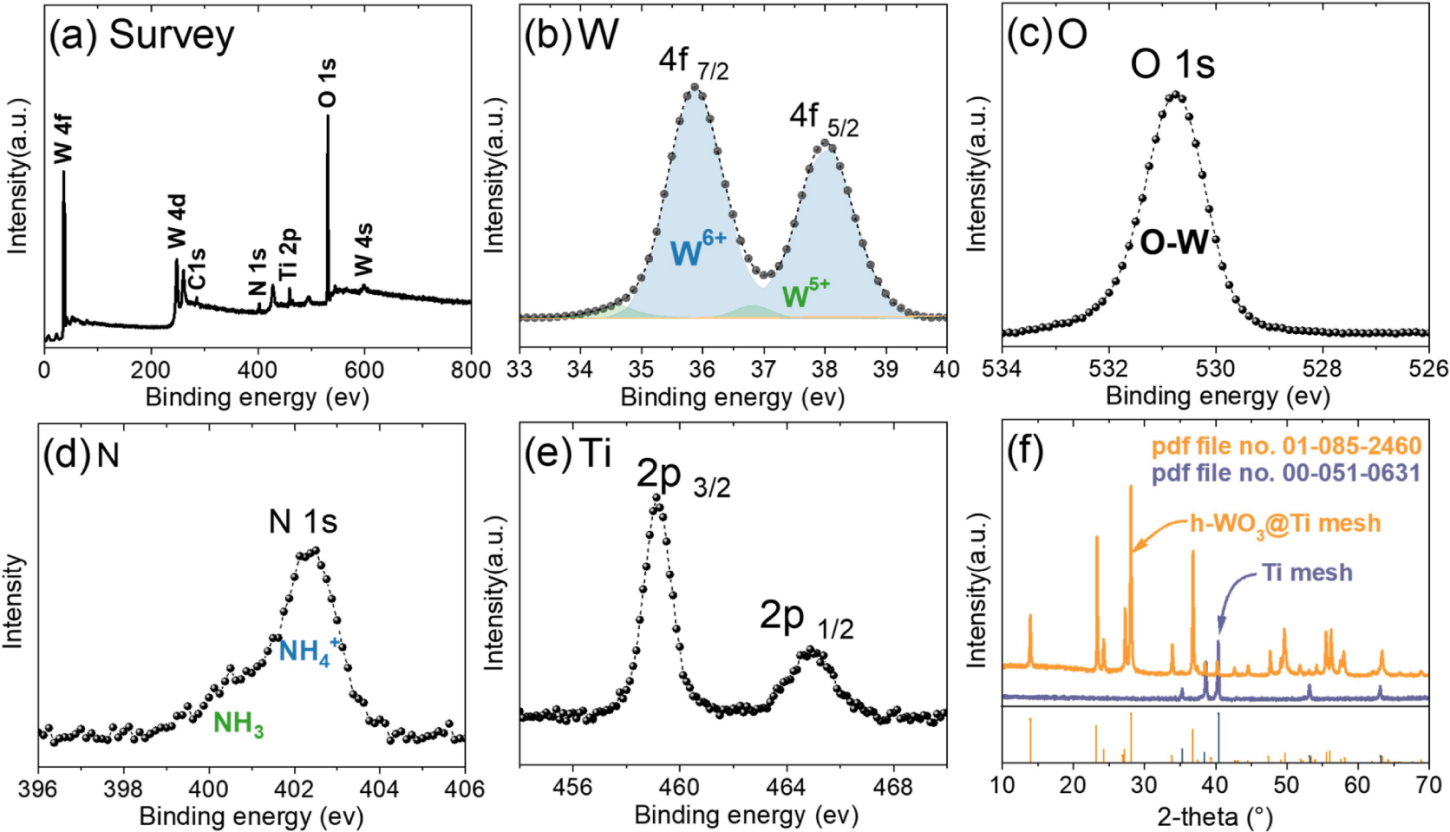
Chemical and microstructure characterization of prepared h-WO_3_@Ti: (a) XPS survey spectrum, (b) W, (c) O, (d) N, (e) Ti spectra, and (f) XRD patterns of h-WO_3_ and Ti mesh with corresponding reference patterns.

The survey spectra (Figure 2a) of prepared h-WO_3_ show distinct peaks corresponding to W 4f, O 1s, N 1s, and Ti 2p, respectively. The W 4f spectra show two sets of binaries with deconvoluted peaks with varying intensities (Figure 2b). The first set of peaks at binding energies of 35.87 and 38.00 eV reveals the high intensity and predominant peak area under a curve of 95% corresponding to W^6+^. In contrast, the other set of two peaks revealed very low intensity and area under the curve at binding energies of 34.62 and 36.87 eV, which correspond to W^5+^. W^5+^ represents the defects of the h-WO_3_ surface, indicating the presence of oxygen vacancies.^27^ Figure 2c represents the O 1s spectra, and Figure 2d represents the N spectra, displaying the presence of NH_4_^+^ ions in the hexagonal channels of h-WO_3_. The presence of NH_4_^+^ ions enables the stabilization of h-WO_3_ and is also a key factor in controlling the shape of nanorods. Specifically, NH_4_^+^ ions preferentially adsorb on the {001} facet, inhibiting crystal growth along this axis.^27^ The Ti spectra exhibit deconvoluted peaks at binding energies of 459.12 and 464.87 eV corresponding to Ti^4+^ (Figure 2e).

The XRD patterns of WO_3_ powder as well as WO_3_ grown on different substrates like Ni and Ti are represented in Figure 2f and S2. The XRD pattern for the Ti substrate and prepared material deposited on the Ti mesh with characteristic reflections corresponding to h-WO_3_ is shown in Figure 2f (reference pattern identified by the PDF file number 01-085-2460 for h-WO_3_).^28^ WO_3_ serves as an excellent model material due to its ability to crystallize into multiple open structures, with the most notable being monoclinic WO_3_ (m-WO_3_) and h-WO_3_ polymorphs. Both forms are composed of WO_6_ octahedra but differ in how they are connected. In h-WO_3_, the WO_6_ octahedra form six-membered rings by sharing corner oxygens along the (001) plane, creating rigid tunnels that extend along the *c*-axis throughout the unit cell.^28^ The Ti mesh shows a distinct set of reflections corresponding to the pure Ti phase (reference identified by the PDF file number 00-051-0631).^29^ The diffraction reflections of Ti are sharp and well-defined, indicating the crystalline nature of the Ti metal. However, the XPS spectrum of Ti corresponds to Ti^4+^, while the XRD pattern indicates the presence of the Ti metal. This discrepancy may arise from the WO_3_ interaction with Ti through oxygen bridges, suggesting that only the interfacial surface of Ti is affected by the hydrothermal growth process, and another reason may arise because XPS can only probe depths of down to 10 nm (electrons’ ability to penetrate material is limited), whereas XRD can penetrate much deeper, reaching depths of up to several hundreds of micrometers (depending on the absorption coefficient). Furthermore, the reflections of the pure Ti metal are present for the h-WO_3_@Ti pattern, showing the presence of Ti beneath the deposited h-WO_3_ without involvement in any chemical interaction. On the other hand, the h-WO_3_@Ni mesh/foam patterns are compared with the powder sample (Figure S2), and all the reflections are aligned to the h-WO_3_ phase in addition to the reflections of the Ni metal substrate.^30^ However, from SEM images (Figure S5), other Ni–O–OH deposits appear, but they are X-ray amorphous.

The Raman spectra (Figure S3) of the as-prepared h-WO_3_@Ti nanowires (NWs) are registered as well. However, Raman spectra are very sensitive to the presence of oxygen vacancies.^31^ The prepared h-WO_3_ is found to be pure h-WO_3_. In the Raman spectrum of the hexagonal WO_3_@Ti sample, the main bands at 777.5, 723.1, and 635.5 cm^–1^ are characteristic of the O–W–O stretching vibrations.^31a,32^ Generally, the Raman spectrum of h-WO_3_ nanowires exhibited overlapping bands in the region of 600–800 cm^–1^. Another considerable difference is that exclusively in the Raman spectra of h-WO_3_ NWs, terminal W=O stretching modes are recognizable in the interval of 925–965 cm^–1^. These peaks are common for all types of WO_3_ hydrates, and in some cases, the appearance of these bands is attributed to surface humidity. The XPS spectra (Figure 2c) show only the O–W peaks, confirming the presence of water molecules. Additionally, the bands at 286.5 and 242.1 cm^–1^ are identified as O–W–O bending vibrations. The peak at 172 cm^–1^ is a lattice vibration of the h-WO_3_ structure.^33^ It is worth noting that h-WO_3_ is always in a partially reduced state due to the presence of stabilizing positive ions in its hexagonal channels, which is confirmed by the XPS spectra showing W^5+^ (Figure 2b), as well as the presence of NH_4_^+^ (Figure 2d).^27^

The surface morphology and elemental distribution of WO_3_, both in powder form and self-grown on different substrates, are examined using a scanning electron microscope (SEM) combined with energy-dispersive X-ray spectroscopy (EDX). The SEM images revealed distinct differences between the two morphologies of WO_3_ (Figure 3, S4 and S5).

**Figure 3:**
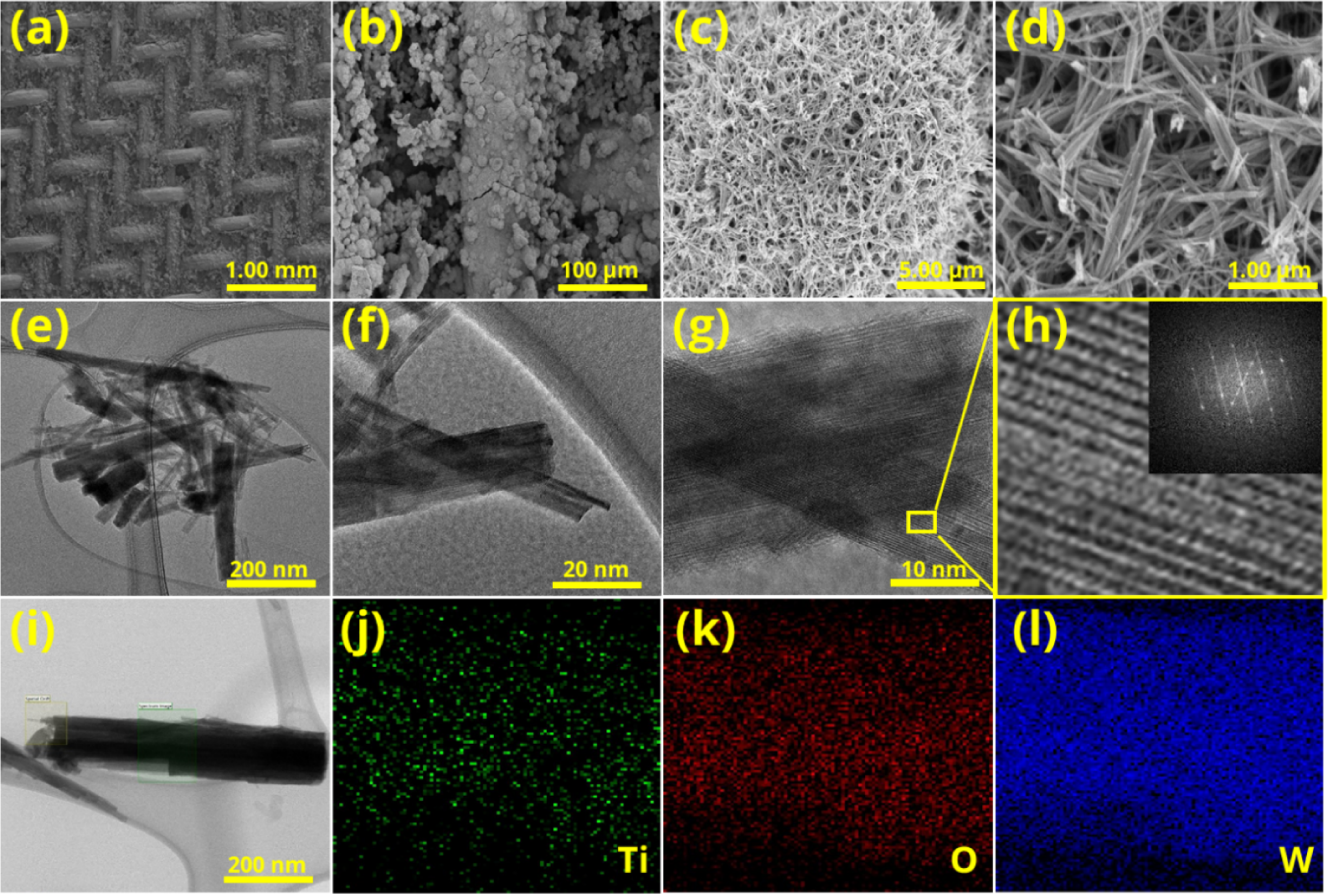
Surface characterization of the prepared h-WO_3_@Ti mesh: (a–d) surface morphology at different magnifications, (e–g) TEM images of peeled h-WO_3_, (h) SAED pattern of the prepared h-WO_3_, and (i–l) elemental distribution of W, O, and Ti elements.

In high-magnification images, the powder form of WO_3_ exhibited agglomerated nanoparticles, forming irregularly shaped particles (Figure S4). In contrast, images of WO_3_ grown on Ni/Ti show a network of nanowires clustered into spherical structures (Figure 3a–d and S5a–d). However, for the h-WO_3_ grown on the Ni foam/mesh, in addition to WO_3_ nanowires, large particles are observed, indicating a rough surface on the Ni substrate. To further investigate the surface composition and elemental distribution, EDX analysis was performed (Figure S6). WO_3_ grown on a titanium mesh primarily consisted of tungsten and oxygen, with traces of carbon and nitrogen, likely residuals from the hydrothermal treatment. In contrast, SEM images of the Ni substrate revealed large surface particles, which EDX confirmed to be NiO/Ni­(OH)_2_. The uniformity and purity of WO_3_ on the Ti mesh render it as the preferred material for electrochemical characterization, and therefore, it will be used for further device optimization. Additionally, elemental mapping showed a homogeneous distribution of W and O across the Ti mesh surface, highlighting the effectiveness and efficiency of the growth process.

To gain a more comprehensive understanding of the crystallinity and structure of WO_3_@Ti and WO_3_@Ni, peeled-off partitions of both were analyzed using transmission electron microscopy (TEM) (Figure 3e–g and S5e–g). In WO_3_ grown on the Ti mesh, the TEM images confirmed a highly crystalline nanowire. In contrast, WO_3_ grown on the Ni foam substrate displayed small, highly crystalline WO_3_ particles, alongside larger amorphous particles. These larger particles that shrink under the electron beam are indicative of NiO/Ni­(OH)_2_. This observation also explains the absence of NiO/Ni­(OH)_2_ reflections in the XRD patterns, as the amorphous nature of the material does not produce sharp diffraction peaks. The results in Figure 3h and S5h show the selected area electron diffraction (SAED), and the fitted results revealed that the lattice fringes mainly correspond to the (101), (002), (102), and (111) lattice planes of hexagonal WO_3_ (PDF file number 01-085-2460). The TEM elemental mapping (Figure 3i–k) shows a uniform distribution of W and O elements for the h-WO_3_@Ti mesh and h-WO_3_@Ni foam sample. The large particles represent amorphous NiO/Ni­(OH)_2_ (Figure S5i–k).

The surface, elemental, and chemical composition analyses confirm the successful synthesis of WO_3_, with its morphology, crystallinity, and purity meeting the desired features. Among the substrates evaluated, the Ti substrate demonstrated excellent characteristics, including high chemical resistivity during hydrothermal treatment. This stability makes Ti an ideal substrate for WO_3_ deposition as it prevents any undesired interactions that could alter the properties of the WO_3_ layers. In contrast, the Ni substrate exhibited reactivity during the same process, leading to the formation of NiO/Ni­(OH)_2_ alongside WO_3_. This secondary phase limits the utility of Ni as a substrate for WO_3_ iontronic application, as the formation of NiO/Ni­(OH)_2_ could degrade the performance and purity of the WO_3_ layer. Therefore, Ti substrates are preferred over Ni for applications requiring stable WO_3_ coatings, particularly in hydrothermal environments.

### CAPode Performance

The electrochemical behavior of WO_3_ deposited directly on different substrates was tested in a volume cell by using a three-electrode configuration (Figures S7 and S8). A platinum electrode (Pt mesh; 3 × 4 cm) was employed as the CE and Ag/AgCl (with a 3 mol L^–1^ KCl internal electrolyte) as the reference electrode (RE). The performance of WO_3_ on different substrates, namely, Ti (@Ti), carbon cloth (@CC), and Ni (@Ni) mesh, was evaluated, along with a WO_3_ powder sample as a free-standing electrode (@SS) composed of an 80:15:5 ratio of active material, conductive additive, and binder, respectively (Figure S9). In general, the current response of WO_3_@Ni was not stable in the selected potential window in contrast to WO_3_@Ti showing high stability across various potential windows. Additionally, the CV curve of WO_3_@Ni displayed a higher and broader oxidation peak compared with the reduction peak, which may be attributed to the oxidation of the Ni substrate. In addition, the CV curve of the WO_3_@CC electrode revealed a broader redox peak extending to the positive polarization region compared to other samples. The higher surface area of CC, compared to the metallic mesh, leads to localized deposition of WO_3_ on the outer surface of CC, leaving uncoated sections exposed to the electrolyte. This effect led to a high capacitance under blocking polarization due to a high surface area for EDL formation.

In contrast, previous studies^15a,18^ have shown that the Ni foam substrate displayed promising performance, although its use as a working electrode (WE) is limited in acidic media. The h-WO_3_@Ti mesh exhibited promising results, demonstrating a steady performance in acidic media up to 2 V without electrolyte decomposition. This stability is advantageous for manipulating potential shifts in G-Caps.^13^ In acidic environments, NiO/Ni­(OH)_2_ tends to dissolve due to the reaction with H_3_O^+^, forming soluble Ni^2+^ and H_2_O in the reactions given in eqs 1 and 2.

1 $$\mathrm{NiO}+2H_{3}O^{+}\to {\mathrm{Ni}}^{2+}+{3H}_{2}O$$

2 $$\mathrm{Ni}{\left(\mathrm{OH}\right)}_{2}+{2H}_{3}O^{+}\to {\mathrm{Ni}}^{2+}+{4H}_{2}O$$

This reaction leads to the gradual dissolution of NiO/Ni­(OH)_2_, rendering it unstable under acidic conditions and reducing the voltage window for this composite. It is also important to note that free-standing electrodes are not ideal because the combined conductive additives such as carbon nanotubes (CNTs) or carbon black may also electrosorb ions under “blocked” polarization, leading to a capacitive contribution to the current response and decreasing the rectification ability of the designed devices.

WO_3_@Ti was evaluated as an electrode in various electrolytes, including 0.5 mol L^–1^ H_2_SO_4_, PVA/H_2_SO_4_, Li_2_SO_4_, Na_2_SO_4_, K_2_SO_4_, and Mg_2_SO_4_ using the CV technique (Figure S4).

In the potential window around 0 V *vs.* RE, the current response from the CV curves of the h-WO_3_@Ti shows two distinct peaks in all electrolytes, indicating that two successive intercalation steps occur under negative potential *vs*. RE (eq 3). The reverse reaction reveals two distinct peaks for the deintercalation steps, confirming the effective reversibility of the prepared material.^34^

3 $${\mathrm{WO}}_{3}+{xH}^{+}+{xe}^{-}\to H_{x}{\mathrm{WO}}_{3}$$

Additionally, the current response of h-WO_3_@Ti in all electrolytes is compared in Figure S10. Generally, electrolytes with smaller cation sizes such as H_2_SO_4_ show a higher current response (Figure S10a) than those with bigger cation sizes, such as MgSO_4_ (Figure S10d). The ion size and charge of cations such as H^+^, Li^+^, Na^+^, K^+^, and Mg^2+^ play a crucial role in the intercalation behavior of h-WO_3_. Smaller ions like H_3_O^+^ (100 pm) and Li^+^ (76 pm) can diffuse quickly and easily within the h-WO_3_ lattice,^35^ allowing efficient and reversible insertion and extraction. This property enhances the performance of systems like lithium-ion batteries, as it minimizes structural strain and supports high cycling stability.^36^ In contrast, larger cations such as Na^+^ (102 pm) and K^+^ (138 pm) introduce more significant lattice distortion during intercalation, which can slow diffusion and compromise long-term structural integrity. Divalent cations like Mg^2+^, although similar in size to Li^+^, have higher charge densities, leading to stronger electrostatic interactions that may cause lattice strain and higher activation barriers for ion diffusion in the solid state.^37^ As a result, the mobility of these larger or higher-charged ions is generally lower in the lattice, impacting the durability and efficiency of the material in applications requiring rapid ion mobility and cycling stability. For this study, H_2_SO_4_ was selected for further device optimization. Additionally, the current response of h-WO_3_@Ti is tested in a more positive window up to 2 V *vs*. RE, and the CV shows that no significant current is observed, indicating high stability (Figure S10). Furthermore, the electrochemical behavior of the Ti substrate was tested in 0.5 mol L^–1^ H_2_SO_4_, and the CV curve revealed solvent decomposition occurring at approximately −0.2 V *vs.* RE (Figure S10e).

The h-WO_3_@Ti electrode serves as the WE in the construction of the CAPode device, with NC@Ti functioning as the CE. The performance of the CAPode is initially investigated using the CV technique at a constant scan rate of 20 mV s^–1^ while varying the voltage window (Figure S11). Basically, the CAPode concept is fundamentally based on an asymmetric current response at around 0 V, mimicking a semiconductor diode. It exhibits a high current response under specific polarization conditions, referred to as “open/chargeable” polarization (in this case, “negative” voltage), and a low current response known as “blocked” polarization (in this case, “positive” voltage).

The voltage window of the designed CAPode plays a crucial role in the application of CAPode as a core for the G-Cap system. Therefore, the voltage windows are tested symmetrically around the switching point (zero voltage; Figure S11). All tested systems show excellent prospective to be used as CAPode even in a voltage window reaching ±2.2 V. Although the CV curve shapes of all tested CAPode systems are very promising under a low voltage window, increasing the voltage window leads to an increase in the current response in “blocked” polarization due to electrolyte decomposition limiting the usage of these electrolytes.

The rectification ratios (RR_I_ and RR_II_; eqs 8 and 9, respectively), measuring the ability of the CAPode to rectify the current response under “open” and “blocked” polarization, are considered as the main criteria controlling the applicability of the CAPode as an iontronic device. In addition to the aforementioned factors, the current at 0 V is considered an additional factor influencing the logic operation. In this regard, the rectification ratios of the constructed system in different electrolytes are calculated at different scan rates, and the results are depicted in Figure S12. The CAPode with 0.5 mol L^–1^ H_2_SO_4_ demonstrates the highest RR_I_ and current response under “open” polarization. Specifically, this system exhibited an impressive RR_I_ = 58, indicating excellent rectification. As a result, it will be used for further investigations of the constructed system. In comparison, RR_I_ of other electrolytes, such as Na_2_SO_4_ and MgSO_4_, also take high values of 18 and 13, respectively. Furthermore, the rectification ratios calculated for different electrolytes have been compared with values reported in the literature (Table S1). It is worth noting that RR_I_ and RR_II_ decrease with increasing the scanning rate. At low scan rates, the electrochemical reaction is diffusion-controlled, giving ions sufficient time to diffuse into and out of the WO_3_@Ti electrode. This enables complete intercalation, enhances charge storage, builds up polarization effectively, and maximizes rectification ratios. The switching point remains stable, ensuring a pronounced difference between open and blocked polarization. In contrast, at high scan rates, the system becomes kinetically controlled where ion movement is limited by diffusion rather than intercalation. Charge storage shifts toward surface adsorption, reducing capacitance and polarization effects. As a result, the switching point shifts due to insufficient equilibration, leading to a more symmetric current response and lower rectification ratios.

The introduced CAPode device demonstrates an outstanding rectification efficiency compared to previously reported CAPode devices, achieving an RR_I_ of 58 and an RR_II_ of 95%. Only Ma et al. reported a CAPode device with a rectification ratio of 135 for RR_I_, but a different calculation method was used.^11c^ Specifically, their calculation involves the ratio between the summation of absolute charging and discharging currents under “open” polarization at the same time and the current summation under “blocked” polarization. While this represents an alternative approach to calculate the rectification of a CAPode device, including both charging and discharging currents simultaneously does not provide a direct indication of the device’s actual rectification performance.

The construction of the CAPode system was optimized in detail based on the mass ratio between WE/CE (Figure S13), electrolyte type and concentration (Figure S14), and the appropriate substrate. The optimized system exhibits excellent switching performance from low current to high current at all tested scan rates, with a consistent switching point around –0.5 V. The significant “negative” voltage shift at –0.5 V under open-circuit polarization highlights the device’s ability to minimize current at 0 V (Figure 4a).

**Figure 4:**
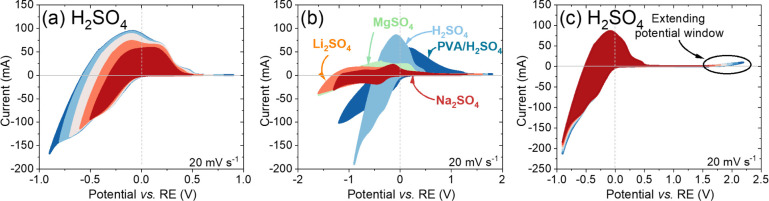
(a) CV curves of h-WO_3_@Ti at different potential windows in 0.5 mol L^–1^ H_2_SO_4_. (b) Comparison of CV curves in the maximum applied potential window of Li_2_SO_4_, Na_2_SO_4_, K_2_SO_4_, MgSO_4_, and H_2_SO_4_. (c) CV curves registered for the h-WO_3_@Ti electrode in 0.5 mol L^–1^ H_2_SO_4_ with window extension in a positive direction of potentials.

The GCD profiles (Figure 5b) support the findings of CV, which demonstrate that the device’s capacity under “positive” voltage is significantly lower compared to “negative” voltage. The charging and discharging time of the system under “negative” voltage is 175 s at 1 A g^–1^, whereas only 0.5 s is required for charging and discharging under “blocked” polarization, confirming the excellent capacity rectification of the proposed system. The associated rectification ratios of the device are calculated and displayed in Figure 5c. Significant RR_I_ and RR_II_ values of 58.7 and 95% (at 2 mV s^–1^), respectively, are achieved, confirming the diode-like characteristics of the device and its ability to rectify current while maintaining energy storage properties. Furthermore, for analyzing the ability of the device to store energy only under a “negative” voltage, the reaction mechanism of the device is tested through *in situ* electrochemical measurements. In addition to the WE/CE in the CAPode device, a RE was added to simultaneously monitor the potential of both the WE and CE electrodes during the charging and discharging process (setup Figure S7c). The black dashed line represents the device’s voltage response (GCD profile), while the red and blue lines represent the potential distribution of the WE and CE, respectively, from OCV (−0.5 V) to −1 V (Figure 5d) and from OCV (−0.5 V) to −1.5 V (Figure S15a). Additionally, to further confirm the mechanism (intercalation steps), the scan rates were calculated based on the rate of potential changes during the discharge step, and the CV technique was employed separately for WE and CE (in the 3-electrode setup; Figure S7a) to investigate the current response of both electrodes (Figure 5e). The CV curve (red line) for WE shows that only one intercalation/deintercalation step takes place when −1 V is applied. However, when −1.5 V is applied, two intercalation steps are observed (Figure S15b). Interestingly, the first-stage behavior of the WO_3_ electrode appears to align more closely with computational studies conducted by Ozoliņš et al., who proposed that proton conduction within WO_3_ occurs via bridge oxygen atoms, rather than through the Grotthuss mechanism.^38^ This distinction suggests that proton migration in WO_3_ might follow a pathway distinct from traditional water-mediated diffusion, emphasizing the role of the material’s intrinsic lattice structure in facilitating ionic conduction.^22^ The rectangular shape of the CV curve registered for CE indicates a typical capacitive charge storage mechanism (EDL formation), regardless of the voltage applied to the system. Furthermore, *in situ* tests were conducted by incorporating an RE into the operating CAPode to monitor the potential distribution of both the WE and CE in a voltage window of ±2 V (Figure S15c). The results confirm that the introduced CAPode can be operated at up to ±2 V without triggering water decomposition. However, extending the voltage beyond this limit led to water electrolysis. The *in situ* measurements further demonstrate that both the WE and CE operate within a safe potential range relative to the electrolyte, without exhibiting signs of water decomposition.

**Figure 5:**
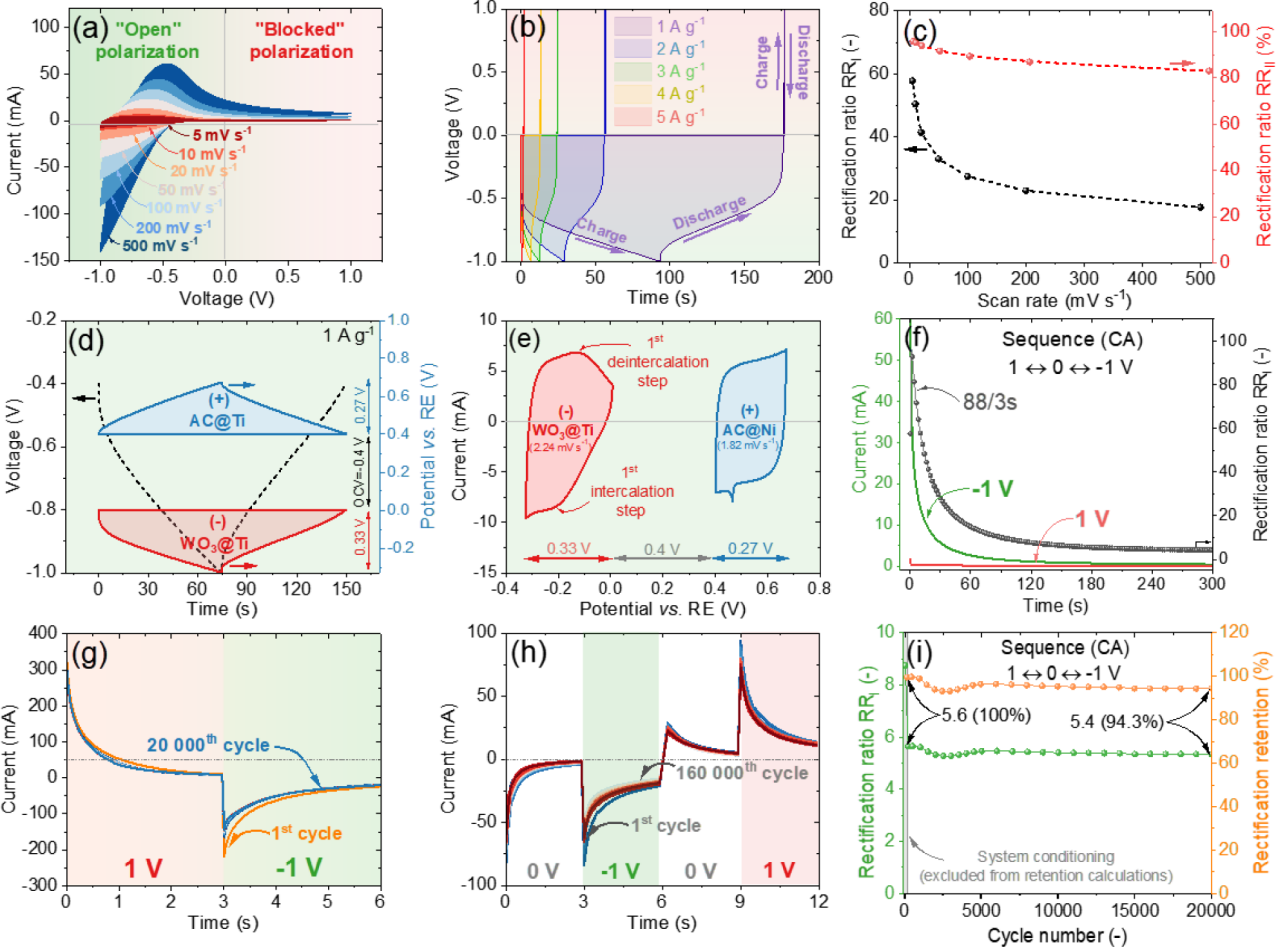
Electrochemical performance of WO_3_@Ti|0.5 mol L^–1^ H_2_SO_4_|NC@Ti: (a) CV curves at different scan rates. (b) GCD profiles at different current densities. (c) Calculated RR_I_ and RR_II_ of the optimized hybrid system at different scan rates. (d) GCD profile (black dashed line) of the system in the range of OCV ↔ −1 V, with simultaneous monitoring of the electrochemical potential distribution of the WE and CE (red and blue lines, respectively). (e) CV curves of the WE and CE at scan rates of 2.24 and 1.82 mV s^–1^, calculated from the GCD profile. (f) The *I*–*t* profile of the system for 300 s using the CA technique with a −1, 0, and 1 V sequence. (g) The *I*–*t* profile of the system under ±1 V, for 3 s each, without a rest step for 20,000 cycles. (h) The *I*–*t* profile of the system with a 3 s pulse period under ±1 V with a rest step for 160,000 cycles, and (i) calculated RR_I_ and rectification retention over 20,000 cycles for the system with a rest step of 3 s.

Beyond the *I*–*U* measurements, an additional analysis was performed to test the device’s switchability. The switching performance of the optimized system was tested under various conditions and extended cycles, up to 20,000 cycles. For the long-term chronoamperometry (CA) pulse test, several sequences were designed to observe the system’s current response under varying conditions. First, Figure 5f shows the current response of the system under a ±1 V pulse with a 0 V resting step (300 s for each step). The results show that when a −1 V pulse is applied, a high current response is initially achieved, which gradually decreases over time, reaching stability after around 300 s. The initial surge in current is attributed to proton intercalation into hexagonal channels and the reduction of W^6+^ to W^5+^ in the first phase, followed by a further reduction to W_4_+, which explains the prolonged change in current. In contrast, when a 1 V pulse is applied, the current response is minimal from the beginning and remains stable for 300 s. Under “blocked” polarization, the WE becomes positively polarized, attracting SO_4_^2–^ ions to its surface. Due to the relatively bigger size of this ion compared to the proton, combined with tungsten already being in its highest oxidation state (W^6+^), no significant current is generated from intercalation or redox reactions. This explains the minimal current under “blocked” polarization. The calculated RR_I_ takes a higher value at the beginning (after 3 s it is 96), but decays over time. This is likely due to surface saturation and continuous reactivation. The slight decay in the current response over prolonged cycling can be attributed to the reactivation of the CE, particularly in aqueous electrolytes. *In situ* long-term cyclic stability tests conducted in a volume cell with a RE reveal that under negative polarization (“open” polarization), the potential distribution of both the WE and CE remains highly stable, ensuring balanced charge dynamics between intercalation at the WE and EDL formation at the CE (Figure S16). However, under positive polarization (“blocked” polarization), a gradual shift in potential occurs due to the increasing capacity or surface activation of the CE, leading to the overpolarization of the WE to maintain charge balance. This effect suggests that the observed slight decay is primarily linked to CE reactivation rather than structural degradation of the WE, as further supported by the stable potential distribution under open polarization conditions.

Additionally, Figure S17a represents another testing condition where a 1 V pulse is directly applied after a −1 V pulse, and Figure S17b represents conditions where a 0 V pulse is directly applied after a −1 V pulse. The results show that when there is no resting step between the voltage switching, RR_I_ is lower. This can be attributed to the desorption of protons from the hexagonal channels and the oxidation of tungsten (W^5+^) to a higher oxidation state under a 1 or 0 V step. Since the intercalation current dominates in this scenario, the switching process is very rapid and the system quickly reaches a steady state. The absence of a resting step reduces the time for proton diffusion and recovery, leading to faster transitions between oxidation states and lowering the overall RR_I_.^15a^

Figure 5g,h represents the short CA pulses (3 s) under a 2- and 4-step sequence, where 0 V is applied before each polarization step for the 4-step sequence. Over the course of 20,000 cycles (Figure 5g) and 160,000 cycles (Figure 5h), the current response remains almost identical to the initial current, demonstrating robust performance over time with minimal degradation or aging in the CAPode. RR_I_ calculated from the CA measurements (Figure 5i and S18) remains nearly constant. The system retains 95% of its initial RR_I_, regardless of the sequence used, confirming its high stability and durability, even after prolonged cycling. This consistency in performance highlights the system’s strong resistance to wear and long-term operational reliability. Additionally, after 20,000 cycles, the surface of the tested sample is analyzed using SEM-EDX (Figure S19). The results show that the surface morphology and elemental composition remained unchanged after long-term cycling.

Structural changes during the charging and discharging processes of the CAPode device were studied in detail. The phase transitions of the h-WO_3_@Ti electrode were tracked using *ex situ* XRD measurements, where the crystal structure was monitored after different charging and discharging conditions (Figure 6).

**Figure 6:**
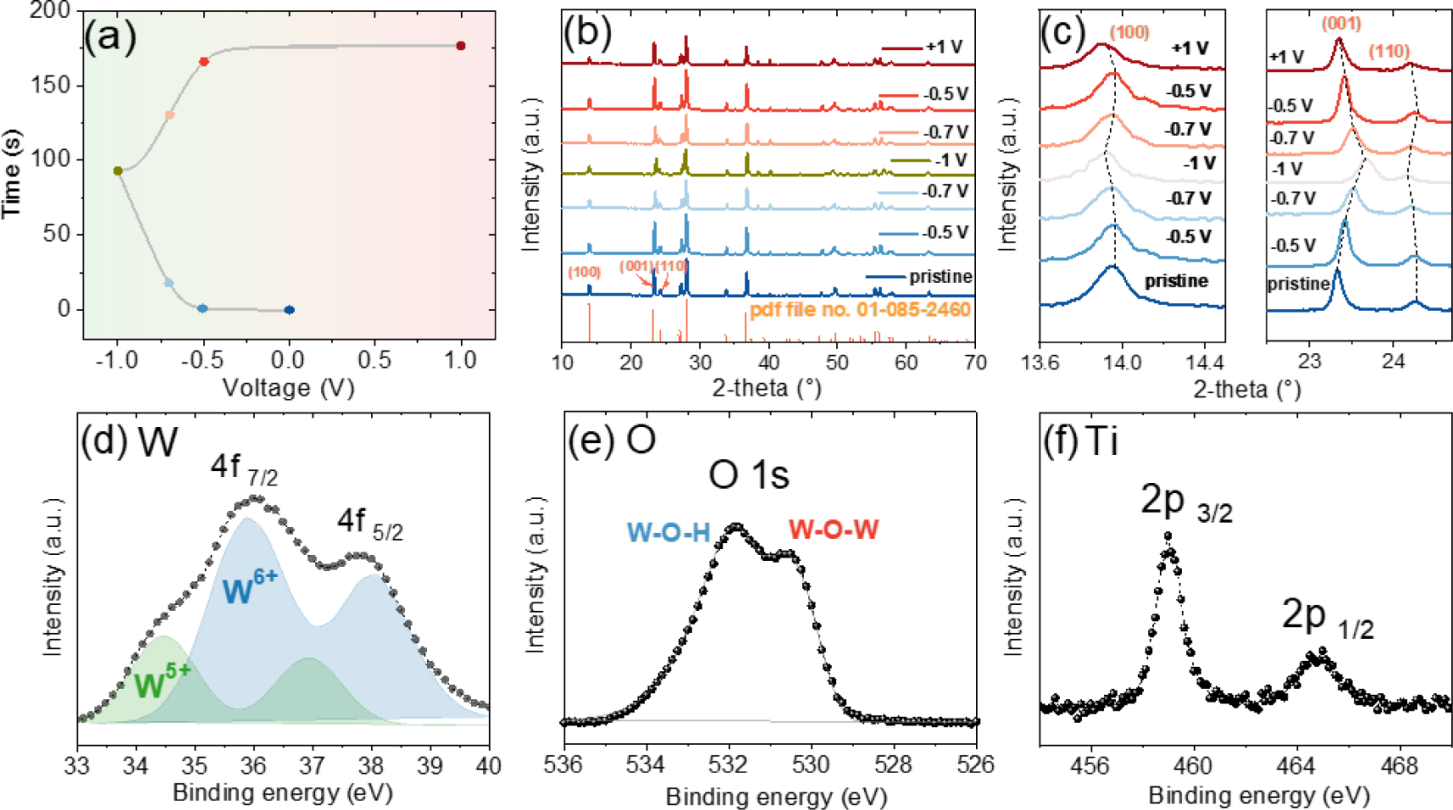
(a) GCD profile of the CAPode device showing testing conditions. (b) *Ex situ-*XRD patterns of the h-WO_3_@Ti electrode under various charging/discharging conditions. (c) Shifts of peak positions under varying voltage. (d) W, (e) O, and (f) Ti spectra of h-WO_3_@Ti after charging at −1 V for 15 min.

*Ex situ*-XRD was employed to track the phase changes during the charge/discharge cycle of the CAPode device. The procedure involved constructing the device in a volume cell, with charging steps conducted using the GCD technique. Measurements were held at specific voltages for 30 min before examination, including −0.5, −0.75, and −1 V for charging and −0.75 and −0.5 V for discharging. Additionally, a voltage of 1 V was applied to test structural changes under “blocked” polarization. Figure 6a shows the GCD profile of the h-WO_3_@Ti electrode, with dotted points indicating the charging or discharging stages where XRD measurements were taken. The XRD patterns (Figure 6b) revealed no significant changes in the phase of h-WO_3_@Ti, although certain planes exhibited notable shifts in their 2θ values, particularly the (100), (110), and (001) peaks. These shifts were attributed to the intercalation and deintercalation of H^+^ ions, which altered the *d*-spacing (Figure 6c).^22^ Figure 6c highlights changes observed in the (100), (110), and (001) planes under various charging and discharging conditions. The shifts became more pronounced at −0.75 V and reached their maximum at −1 V. When discharged, the peaks reverted to their initial positions, demonstrating the reversible nature of the structural changes.

The shifts in diffraction peaks for the (100) and (110) planes to lower 2θ values during charging can be attributed to lattice expansion within the ab-plane caused by H^+^ ion intercalation. As protons are introduced into the crystal structure, they interact with the oxygen atoms within the WO_3_ framework, leading to localized distortions and a slight elongation of the lattice in the a,b directions. This expansion increases the interatomic distances in the ab-plane, causing the *d*-spacing of the (100) and (110) planes to increase, and correspondingly lower 2θ values, resulting in the observed shifts.^22^ In contrast, the (001) plane exhibits a shift to higher 2θ values during H^+^ intercalation. This is due to the contraction of the interlayer spacing along the *c*-axis, as H^+^ ions occupy interlayer spaces and form electrostatic interactions with the oxygen framework such as W–O–H bonds. This contraction reduces the *d*-spacing of the (001) plane, leading to an increase in the 2θ angle. Upon discharging, the deintercalation of H^+^ ions reverses these structural changes, with the (001) plane shifting back to lower 2θ values as the interlayer spacing is restored and the (100) and (110) planes returning to their initial positions as the lateral lattice relaxes. These reversible shifts in 2θ values reflect the dynamic structural changes of h-WO_3_ during the electrochemical process, showcasing its ability to accommodate ionic transport while maintaining structural integrity. The oxidation state of the h-WO_3_@Ti electrode was analyzed after being charged at −1 V for 15 min. The W spectra (Figure 6d) displayed two deconvoluted peaks corresponding to W 4f_7/2_ and W 4f_5/2_. In the pristine sample (Figure 2), only a small amount of W existed as W^5+^. After charging to −1 V, the intercalation of H^+^ ions resulted in the reduction of W^6+^ to W^5+^. Additionally, the O spectra (Figure 6e) revealed two distinct peaks associated with W–O–W and W–O–H bonds, confirming the observed electrochemical behavior. In contrast, the Ti spectra (Figure 6f) showed no changes during the charging or discharging processes, indicating the structural stability and nonparticipation of the Ti framework to overall performance. The XPS spectra of the h-WO_3_@Ti electrode, examined before and after electrochemical polarization, confirm electrochemical intercalation. In the pristine WO_3_, tungsten predominantly exists in the W^6+^ oxidation state, with a minor presence of W^5+^ (4.9%) attributed to synthesis-induced defects. After CAPode polarization at −1 V, the XPS spectra reveal an increase in W^5+^ to 37.1%. These redox-induced changes suggest that only a fraction of the WO_3_ material participates in electrochemical reactions. This active fraction is influenced by factors such as electrode morphology, porosity, and the accessibility of the electrolyte to the redox-active sites.^30^

The XRD peaks of WO_3_@Ti after cycling appear sharper compared to those before cycling (Figure S19), suggesting enhanced crystallinity and defect healing through cycling. The phenomena of XRD reflection sharpening after electrochemical cycling, particularly in certain materials such as layered or intercalation-type compounds, suggest an enhanced crystallinity due to structural ordering and defect healing. The reversible ion insertion-extraction process drives the rearrangement of ions and structural adjustments, which can reduce defects, enhance atomic ordering, and improve lattice coherence. Additionally, relaxing or realigning structural imperfections further contributes to improved crystallinity. As a result, the observed sharper XRD reflections serve as a clear indication of enhanced structural order and defect healing. These analyses further support the electrochemical findings, confirming the stability of the system and the minimal impact of cycling on its structural and compositional integrity.

The Staircase Potential Electrochemical Impedance Spectroscopy (SPEIS) technique was employed to investigate the impedance behavior at the electrode–electrolyte interface under both “open” and “blocked” polarization (Figure S20). When SPEIS measurements were performed under “open” polarization (Figure S20a), a gradual decrease in charge transfer resistance was observed with a voltage increase (more “negative” voltage) as indicated by the smaller semicircle at high frequencies. The resistance reached a minimum value at −1 V. In contrast, under “blocked” polarization (Figure S20b), the internal resistance remained nearly constant, with no significant changes. This behavior is attributed to the rapid EDL formation, as no significant electrochemical reactions occur. When the WE is positively polarized, overpolarization primarily occurs to balance the charge of the CE without initiating any reaction. These observations are further supported by the Bode plots (Figure S20c,d). Under “open” polarization, the total system resistance progressively decreases with the voltage (more “negative” values). Initially, the impedance shows a significant change up to −0.5 V, after which the variations become less pronounced due to the onset of intercalation and redox reactions at this potential, confirming the Nyquist plot results. Conversely, under “blocked” polarization (Figure S20d), the total impedance remains relatively unchanged across the applied voltage range. Furthermore, the phase shift observed in the Bode plot distinguishes the capacitive nature of the electrode surface (Figure S20e, f). Under gradual “open” polarization (Figure S20e), the EDL mechanism initially dominates, with the phase shift reaching approximately 80.75° at 6.31 Hz. As the voltage becomes more “negative”, the phase shift begins to decrease, reaching 27.19° at −0.5 V where intercalation starts to occur. Notably, the phase shift stabilizes at 27.19° under further “open” polarization, indicating continuous intercalation within the applied voltage range of −0.5 to −1 V. At very low frequencies, the phase shift increases again, likely due to the EDL. In contrast, under “blocked” polarization, the phase shift increases from 80.75° to 84.6° at −0.6 V, confirming a purely EDL mechanism. This value remains constant across the different tested voltages. These SPEIS results further validate the described mechanism and align with the current response observed in the CV measurements.

### G-Cap Performance

The optimized CAPode device is integrated with a third GE, transforming it into the core of a G-Cap system.^3,12a^ This system operates similarly to a transistor, where the current flow between the WE and CE can be switched “on” or “off” by controlling the gate electrode. This switching mechanism allows the G-Cap to regulate electrochemical processes, effectively acting as a transistor analogue. In transistor-like systems, the gate electrode plays a crucial role in modulating the flow of charge carriers, allowing the device to either conduct or block current. Two key mechanisms have been proposed for the G-Cap system to achieve this modulation. The first is the ion depletion mechanism, where the gate electrode controls the current flow by either depleting or releasing ions into the electrolyte solution. By altering ion concentrations near the WE and CE, the system can regulate its conductivity, similar to how a transistor’s gate controls electron flow by manipulating electric fields. The second mechanism relies on a potential shift. In this case, the gate electrode controls the current response by shifting the potentials of the WE and CE, toggling between an “open” polarization state (where current flow is enabled) and a “blocked” polarization state (where the current is minimized or blocked).^13^ When the GE is activated, current flow can decrease significantly because the potential region of WE is gradually shifted away from the potential region where redox reactions occur. In previous research systems based on the redox mechanism, the capacity of the proposed system was maintained at 20% of its initial capacity when the gate is switched on, showing that while energy storage is reduced, it is still functional under GE activation.^13^ This highlights the GE’s ability to precisely modulate electrochemical reactions, akin to a transistor’s control over charge flow, opening new pathways for energy management and current rectification in advanced electrochemical devices. Figure 7 illustrates a comparison of the new h-WO_3_-based device responses under different conditions: without bias, under positive bias (1 V), and negative bias (−1 V) .^3,12a^

**Figure 7:**
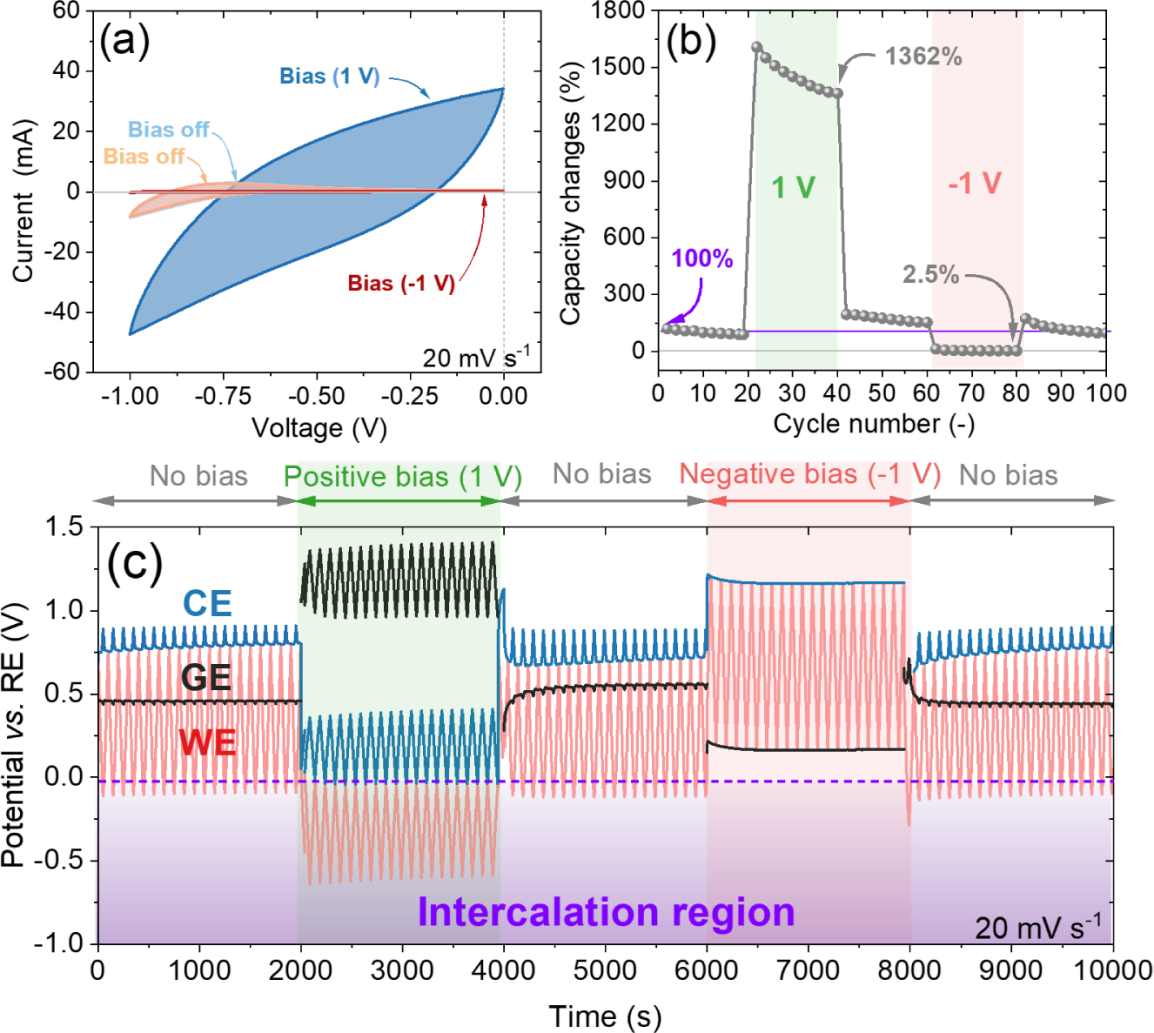
Electrochemical behavior of the G-Cap device: (a) CV curves registered before bias, under positive bias, and negative bias; (b) calculated changes in capacity over 20 cycles for each step; and (c) potential distribution of the WE, CE, and GE during each phase of the process against Ag/AgCl RE.

The device maintains 2.5% of its initial capacity (Figure 7a,b), demonstrating significant control over current rectification. Notably, the device exhibits a 14-fold increase in capacity under reverse bias conditions, suggesting that its capacity depends mainly on the direction of the applied bias. This unique feature opens up new possibilities for rectifying current and controlling energy storage based on external voltage. Figure 7c also shows the potential distribution of the WE, CE, and GE, revealing how each electrode responds under varying GE biases. These potential distribution curves provide insight into the electrochemical processes occurring at each electrode and demonstrate the distinct switching behavior that occurs when a bias is applied. When a positive bias (1 V) is applied, the potentials of the WE and CE shift to more negative values, while the GE electrode shifts to a more positive potential. The potential window of the WE diminishes, while the CE and GE potential windows extend compared to the potential distribution of the electrodes before the bias. This indicates that shifting the WE and CE to more negative potentials increases the capacitance due to an enhanced shift into the intercalation region (−0.05 V), which in turn extends the potential window of the CE and GE to balance the charge of the WE and CE. Conversely, when a negative bias (−1 V) is applied, the potential distribution of the WE extends, while the potential window of the CE and GE becomes minimal. This behavior occurs when the WE moves out of the intercalation region, resulting in a limited contribution to the intercalation capacity and a primary reliance on the EDL capacitance. Consequently, the potential distribution of the CE and GE remains largely unchanged during cycling, while the WE consumes the entire applied voltage and becomes overpolarized to balance the charge of the CE and GE. Additionally, h-WO_3_@Ti has a limited surface area compared to NC (877 m^2^ g^–1^; Figure S21), which leads to overpolarization of WO_3_@Ti to balance the charge accumulated on the NC. The device’s ability to switch capacity and adjust energy storage under different biases introduces a novel approach to designing electrochemical devices.

### Logic Gates

EC diodes operate with ions instead of electrons in semiconductors, mimicking the logic operations of neurons, synapses, and ionic channels in biology. The human brain is evolutionarily highly optimized in terms of low energy consumption. Moreover, the ability to operate with biologically active ions may enable interfacing iontronic systems with biological nervous systems in the future.^2,39^ Thus, ionologic systems promise critical advancements, such as lower computing energy consumption and biocompatibility.

However, one of the critical challenges in developing ionologic gates is the issue of “channel talking” between devices, especially when two devices are coupled together in such configurations.^11,15^ This issue arises from the high current observed during the charging or discharging processes at 0 V, significantly affecting the output signal integrity. Previous attempts to address this problem, such as CAPode systems based on an ion-sieving mechanism, have shown promising results.^11b,16b^ However, “channel talking” still remains a prominent issue in logic gate applications.^11b^ A critical aspect is the interaction of devices during repolarization changes, particularly when carbon-based materials are used for device construction. This interference leads to undesirable output fluctuations. One potential solution is the use of CAPode systems based on redox reactions, which limit high current to specific voltages (i.e., the redox voltage) or have a carbon-based system that switches at “positive/negative” voltage rather than at 0 V. However, conventional methods of constructing working electrodes whether through slurry, hard, or soft fabrication techniques often involve binders and conductive agents and therefore still exhibit the issue of one device consuming a fraction of the output signal of the other device during polarization changes.^15^

In contrast, the proposed approach of using self-grown Ni_3_Bi_2_S_2_ as the WE has demonstrated promising logic gate and electrochemical performance.^15a^ However, its application is limited to ±0.7 V due to electrolyte decomposition, preventing higher voltage application.^15a^ This problem is solved in the new device structure proposed here, featuring h-WO_3_ grown directly on a Ti mesh surface as the WE. This architecture allows operation up to ±2 V without electrolyte decomposition or electrode degradation, ensuring a stable performance. The applied voltage used as inputs for the devices in OR and AND gates plays a critical role in shaping the characteristics of the output signal and ensuring stability. The effects of input voltage and frequency on the performance of simple logic gates are studied with the results shown in Figure 8 and S22–S24.

**Figure 8:**
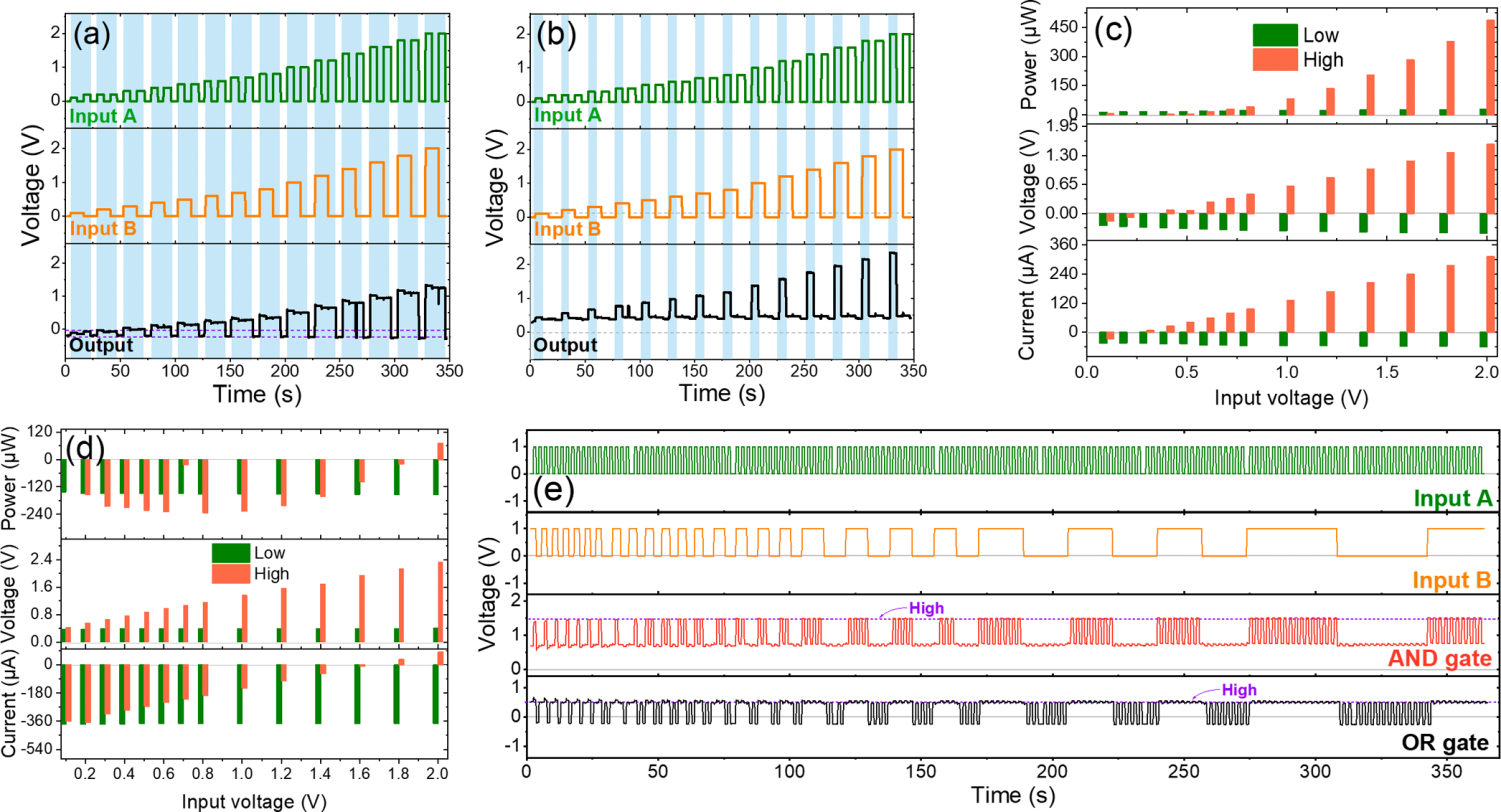
CMOS logic gate results of the constructed devices under various applied voltages: (a) OR gate results when both inputs A and B gradually increase after one complete operation from 0.1 to 2 V with input frequencies of 100 and 50 mHz, (b) AND gate results when both inputs A and B gradually increase after one complete operation from 0.1 to 2 V with input frequencies of 100 and 50 mHz, (c) the corresponding current voltage drop and power of the OR gate for each operation under different input voltages from 0.1 to 2 V, and (d) the corresponding current voltage drop and power of the AND gate for each operation under different input voltages from 0.1 to 2 V. The orange-colored current, voltage, and power represent the output when it is high (1); the green-colored current, voltage, and power represent the output when it is low (0). (e) The switching performance of OR and AND gates under different frequencies between the inputs A and B.

Figure S23 presents the basic OR and AND gate connections alongside the truth table for both gates. For the OR gate, output signals were tested across various input voltages ranging from −0.2 to −2 V (Figure S24). The results demonstrate a performance comparable to that of a commercial diode across all voltages, indicating reliable functionality. However, as the input voltage increased, slight deviations were observed for the (1, 0) and (0, 1) inputs compared to the (1, 1) input. This deviation is likely due to the fact that the device under 0 V can consume a very small part of the current, which decreases the output current, leading to pulling the output voltage to lower value. Figure S25 displays the applied voltage input results for the AND gate. The output signals maintained the expected characteristics of an AND gate across all of the tested input voltages. Small spikes appeared on the output signal when one of the inputs, A or B, was switched, while the other remained constant. These spikes were minimal at lower applied voltages but increased slightly in magnitude with higher applied voltages. Despite the presence of these spikes, they remained very small compared to the overall output signal.

In addition to the input voltage range, the input frequency plays a significant role in determining the potential applications of iontronic devices. Unlike electronic devices, which can operate at high frequencies reaching the megahertz (MHz) range, iontronic devices are limited in frequency due to their electrochemical nature. In electronic devices, the flow of electrons is significantly faster than the flow of ions in iontronic devices, while ions require more time to complete electrochemical interactions or form an EDL. Consequently, iontronic devices typically operate at lower frequencies. Previous studies have shown promising results for simple logic gates operating at low frequencies, such as 10 mHz to 50 mHz, which restricts their application to low-frequency fields.^11,15^ However, this study extends the capabilities of iontronic devices by demonstrating performance at higher frequencies, reaching up to 1000 mHz, a significant milestone in the field. The results of testing low and high frequencies (1/2 to 500/1000 mHz for inputs A and B, respectively) are shown in Figure S26. The OR gate operation at both low and high frequencies consistently replicated the behavior of iontronic diodes, validating the device’s suitability for logic applications. Various input frequencies ranging from 1 to 1000 mHz were tested for the AND gate configuration, as displayed in Figure S27. Across all tested frequencies, including 1000 mHz, the output signals retained the characteristic response of an AND gate, demonstrating a stable performance.

### Logic Operation Metrics and Switchability

For a better understanding of the logic operation and quantifying logic metrics, the current flow in the logic gates, voltage drop, and power consumption were calculated (Figure 8). A sequence of logic operations was conducted continuously over a varying voltage window from 0.1 to 2 V for both the OR and AND gates (Figure 8a,b). The outputs of both gates demonstrated excellent logic functionality across all tested voltages, as reported for basic gates (Figures S24 and S25). For the OR gate, at all measured voltage windows, the current recorded at (0,0) inputs remained almost constant around −50 μA, pulling the output to a negative voltage of −0.3 to −0.4 V. In contrast, for semiconductor diodes, when the inputs are (0,0), the output remains at 0 V since no current flows. However, unlike semiconductor diodes, the CAPode device exhibits a negative current at 0 V, which is attributed to the charge redistribution within the electrodes. Before applying 0 V, the electrodes (WE and CE) hold different charge states. When 0 V is applied, the system reorganizes its charges to achieve equilibrium, inducing a negative current flow, which results in a negative voltage drop across the CAPode-based OR gate. Notably, a 0.35 V voltage drop was observed when a 0.1 V input was applied. When 0.3 V inputs were introduced, the recorded current was 0 A, indicating that the current flow due to the high input equaled the negative voltage. Before reaching 0.3 V, OR gate functionality was evident because the current shifted to a more positive value at the high state than at the low state. Beyond 0.3 V, the current began flowing toward the output, enabling power transfer (Figure 8c). Once the 0.3 V threshold was exceeded, a positive voltage was recorded at the output and the current dissipated across the resistor. The trend of the output current (orange columns) remained consistent at 0 V across all tested voltage windows. Consequently, the power dissipation for the OR gate increased as the applied voltage window expanded. Compared to semiconductor diodes, the CAPode exhibits a 0.35 V voltage drop for power transfer, while traditional semiconductor diodes typically have a forward voltage drop of 0.2 to 0.7 V depending on the type. These results confirm that the CAPode device not only demonstrates logic functionality but also exhibits behavior comparable to that of semiconductor diodes. Furthermore, the power, voltage, and current flow in the AND gate were monitored across different voltage windows (Figure 8d). Unlike the OR gate, the AND gate output shifted by 0.4 V, which is attributed to charge redistribution at 0 V. A 2 V power source (VCC) was used to power the AND gate, highlighting the key distinction between AND and OR gates. For the OR gate, the output is determined by two input voltages (A and B), which control the current flow. In contrast, the AND gate introduces a third voltage source (VCC) in addition to inputs A and B, which induces current flow in the CAPode at (0,0), (1,0), and (0,1). Current flows to the output only when both diodes are conducting (high state). At 0 V, the current flowing through the CAPode is at its maximum, gradually decreasing as input voltages A and B increase.

Notably, the current recorded at 0 V for inputs A and B, along with 2 V VCC (−380 μA), matches the current observed in the OR gate at 2 V inputs (330 μA) plus the baseline current at 0 V (−50 μA). This indicates that the VCC voltage plays a crucial role in regulating the current flow within the circuit. Once inputs A and B exceed 1.8 V, the current starts flowing out of the gate instead of into it. Overall, the presented data highlight the high reliability and efficiency of the CAPode device as an analogue to semiconductor diodes, demonstrating its potential for ionic logic applications. The circuit’s power is calculated using the equation *P* = *IU* for both OR and AND gates across different voltage windows. Current is measured before the output, while the voltage drop across the CAPode is determined based on the minimum voltage required to drive current through the circuit. The voltage drop, power consumption, and power dissipation of both the CAPode and the resistor are recorded. The computed power values for the OR and AND gates are presented in Figure 8c,d.

Figure 8e represents the OR and AND gate outputs when the ratio between input frequencies A and B varies by time to examine the switching response of the CAPodes when the frequency of input B changes continuously. Input A frequency is set at 1000 mHz, and input B frequency varies from 500 to 16 mHz. The gate outputs revealed immediate switching to realize the logic operation without any delay for both gates.

Overall, this investigation, along with further measurements, proves that the device can maintain the characteristic functions of electronic diodes while also exhibiting energy storage capabilities of ECs. The results suggest that iontronic devices can offer broad switching capabilities and meet the requirements for various applications where traditional electronic devices may be limited.

More complex logic gates were constructed by using six devices arranged in three interconnected logic gates. The electronic configuration was designed so that the output signals from two gates would serve as the inputs for the third gate (Figure 9).

**Figure 9:**
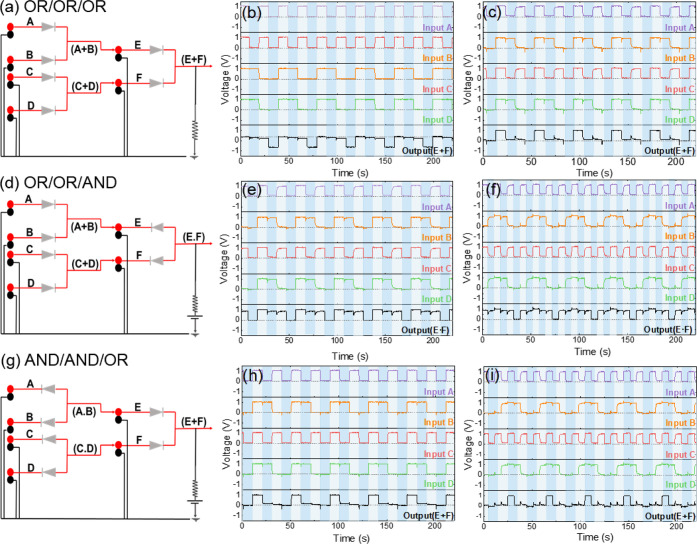
Logic gate results for a complex circuit using three gates with different connection architectures: (a) schematic representation of the electronic connections for the OR/OR/OR configuration, (b) input and output signals registered under the OR/OR/OR configuration, (c) input and output signals observed under the AND/AND/AND configuration, (d) schematic representation of the electronic connections for the OR/OR/AND configuration, (e) input and output signals for the OR/OR/AND configuration with a double frequency ratio between A/C and B/D input signals, (f) input and output signals for the OR/OR/AND configuration with a triple frequency ratio between A/C and B/D input signals, (g) schematic representation of the electronic connections for the AND/AND/OR configuration, (h) input and output signals for the AND/AND/OR configuration with a double frequency ratio between A/C and B/D input signals, and (i) input and output signals for the AND/AND/OR configuration with a triple frequency ratio between A/C and B/D input signals.

The first setup involved creating three OR gates, known as the OR configuration (Figure 9a), and three AND gates, known as the AND configuration (Figure S26). The truth tables for each configuration are listed in Table S2. In the OR configuration, input signals A and B are set to 25 mHz, while inputs C and D are set to 50 mHz (Figure 9b). The outputs from the first two gates, F (outputs of A and B) and E (outputs of C and D), serve as inputs to a third gate. The output signal followed the design of the electronic circuit, matching the expected results outlined in the truth table (Table S2). It was noted that the baseline for the OR connection shifted to a more negative value. Figure S26 shows the output signal for the OR gate when 0 V was applied as the input, indicating a negative shift. A similar shift occurred in the AND gate output but in a positive direction. These shifts are likely attributed to the internal resistance of the electrochemical devices and their connections. Nonetheless, the output signal across the three OR connections aligned with the expected design.

For the AND configuration, the output results are depicted in Figure 9c. The AND gate produced an output of 1 only when all inputs (A, B, C, and D) were set to 1, perfectly matching the truth table. This confirms that the three AND gate connections performed according to the expected logical behavior. Additionally, another configuration involved two OR gates and one AND gate, as illustrated in Figure 9d. In this setup, the output signals of the OR gates served as inputs for the AND gate. The output signals are recorded under different frequency conditions, as shown in Figure 9e,f, and the truth table for the theoretical output expected from the designed circuit is displayed in Table S2. When the input frequency ratios between A and C and between B and D were set to 1:2, the output signal (Figure 9e) matched the theoretical calculations. Similarly, with a frequency ratio of 1:3, the output achieved the expected results (Figure 9f). As the frequency difference between the inputs increased, cross-talk between the channels became more pronounced. This effect is likely due to one of the devices charging and discharging multiple times (three times), while the other devices remained under the same conditions. For another configuration, where two AND gates were connected to an OR gate (Figure 9g), the output signals at different frequencies (Figure 9h,i) also matched the theoretical values outlined in Table S3. Furthermore, the efficiency of constructed devices was further tested under different input frequencies (Figure S27). The constructed logic gates under the configurations (Figure 9a,d,g and S26) were tested under four different frequencies for input signals of 0.5, 0.25, 0.167, and 0.125 Hz. The output signals of all constructed logic gates with different configurations fit theoretical calculations (Table S4). Furthermore, Figure S28 presents the output when both inputs A and B are 0 for OR and AND gates, respectively.

The VCC position in the circuit is a crucial point to operate the AND logic gate. In the case of the AND/AND/AND configuration, the VCC is set after the output gate. Since all gates are in the AND configuration, the current flows in one direction. The current flowing in the output AND gate under this configuration works as a power source for both AND gates in the beginning and continuously supplies current as the VCC is “on” state. On the other hand, the challenge becomes more pronounced when AND and OR gates are connected in one circuit because the current flow in an OR gate is reverse to the current flow in an AND gate. For example, in the AND/AND/OR configuration, the VCC is connected to the OR gate rather than AND gates such as basic AND circuits (Figure S23). This connection positioned the OR gate in the blocking direction. The reason for successfully achieving the logic functionality under this configuration is that when the power supply is connected to the OR gate, a current of ∼−50 μA flows under blocking direction. This reverse current is sufficient to power the two AND gates in the beginning. Furthermore, as noted, the output voltage at (0, 0) inputs could be adjusted to 0 V depending on the CAPode connection in gates. In the basic OR gate (Figure 8a and S24) and AND gate (Figure 8b and S25), the output voltages are shifted by −0.3 V in the OR gate and 0.3 V in the AND gate. The combination of both AND and OR gates together adjusts this deviation, and the output is set to 0 V which is considered as a confirmation to the validity of using the ionic diode as an analogue to semiconductor diodes (Figure 8e,f,h,i). Overall, the proposed CAPode demonstrated behavior resembling that of an electronic diode and efficient integration into interconnected logic gates for ionologic operations.

## Conclusions

The newly developed CAPode and G-Cap devices have demonstrated strong potential as supercapacitor diodes and switchable transistors, showing remarkable stability, highly efficient current rectifications of RR_I_ = 58 and RR_II_ = 95%, and rapid switching capabilities of 97.5%. The rapid H^+^ intercalation and deintercalation into nanostructured h-WO_3_ electrodes are crucial to achieving excellent performance. These characteristics are ideal for ionologic gate applications. Basic logic gate configurations, including AND and OR gates as well as more complex interconnections, were efficiently operated with applied voltages ranging from 0 to 2 V, as well as across a wide frequency range of 1–1000 mHz. Notably, the devices exhibited excellent logic gate functionality, with output signals comparable to those of electronic diodes, even at varying input frequencies. This indicates their capacity for stable and efficient operation across different frequency environments. The output signals closely mirrored those of traditional electronic diodes, marking a significant advancement over previously reported ionic-based CAPode systems.

## Experimental Section

### Materials and Chemicals

Sodium tungstate dihydrate (Na_2_WO_4_ · 2H_2_O), oxalic acid dihydrate (C_2_H_2_O_4_ · 2H_2_O), hydrochloric acid (HCl), ammonium sulfate ((NH_4_)_2_SO_4_), sodium chloride (NaCl), sodium sulfate (Na_2_SO_4_), magnesium sulfate (MgSO_4_), potassium sulfate (K_2_SO_4_), lithium sulfate (Li_2_SO_4_), ethanol (C_2_H_6_O), polyvinyl alcohol (PVA), *n*-methyl-2-pyrrolidone (NMP), polyvinylidene difluoride (PVDF), and Nafion were supplied by Sigma-Aldrich (Germany). Activated carbon SX ultra 8020-3 was supplied by Cabot (USA). All chemical compounds and organic solvents are of analytical grade and can be used without further purification. Nickel (Ni) foam and mesh were obtained from Goodfellow (UK), and Ti mesh was supplied by Guangdong Canrd New Energy Technology Co. (China). The GF/A separator was supplied by Whatman (UK).

### Synthesis Procedure

Working electrodes (WEs) based on nanostructured tungsten trioxide (WO_3_) were prepared using the hydrothermal method as presented schematically in Figure S1. First, 1.86 g of Na_2_WO_4_ · 2H_2_O was dissolved in 50 mL distilled H_2_O and stirred for 20 min until total dissolution (eq 4). Then, 2 mol L^–1^ HCl was added to adjust the pH of the solution to 1.2 (eq 5). After that, 2.2 g of C_2_H_2_O_4_ · 2H_2_O was added under continuous stirring until the solution became clear as a result of complexation between oxalate ions (C_2_O_4_^2–^) and W^6+^ in tungstic acid (WO_3_ · H_2_O), aiding in complete dissolution (eq 6).^1^ A 75 mL of distilled H_2_O was added to the brim solution to reach 125 mL in total, followed by adding 6.29 g of (NH_4_)_2_SO_4_ as a capping agent to prevent agglomeration of particles during thermal treatment. The mixed solution was then transferred to a Teflon-lined holder without and with a substrate. A piece of Ni or Ti mesh was dipped into a mixed solution before being transferred to an autoclave cell to grow h-WO_3_ directly on the substrate surface (Ni foam, mesh, or Ti mesh) (eq 7). The thermal treatment was maintained for 16 h at 160 °C and then gradually cooled at room temperature. The prepared powder/self-grown material over Ti/Ni mesh and Ni foam was washed three times with H_2_O and C_2_H_5_OH and then dried for 24 h at 60 °C.

4 $${\mathrm{Na}}_{2}{\mathrm{WO}}_{4}\cdot {2H}_{2}O\to {\mathrm{2Na}}^{+}+{{\mathrm{WO}}_{4}}^{2-}+{2H}_{2}O$$

5 $${{\mathrm{WO}}_{4}}^{2-}+{2H}^{+}\to H_{2}{\mathrm{WO}}_{4}$$

6 $$H_{2}{\mathrm{WO}}_{4}+H_{2}C_{2}O_{4}\cdot {2H}_{2}O\to H_{2}[{\mathrm{WO}}_{3}{(C}_{2}O_{4})\cdot {3H}_{2}O]$$

$$H_{2}[{\mathrm{WO}}_{3}{(C}_{2}O_{4}){\cdot 3H}_{2}O]+\mathrm{heat}\to {\mathrm{WO}}_{3}+{\mathrm{CO}}_{2}+{4H}_{2}O$$ 7

### X-Ray Photoelectron Spectroscopy (XPS)

The elemental composition of the WO_3_ layers deposited on the Ti substrate was examined, and the oxidation states of the incorporated species were evaluated by using a high-resolution X-ray photoelectron spectrometer (HR-XPS; Thermo Fisher Scientific; USA).

### X-Ray Powder Diffraction (XRD)

The synthesized samples are analyzed using X-ray diffraction (XRD), with patterns recorded on an Empyrean diffractometer (Malvern Panalytical; UK) employing Cu Kα_1_ radiation (λ = 1.54056 Å) and a Ge-monochromator, operating at 40 kV and 40 mA under ambient temperature conditions.

### Raman Spectroscopy

Raman spectra were recorded in a backscattering configuration using a T64000 spectrometer from Horiba Jobin Yvon, with a 660 nm laser excitation source from a Torus Laser (Laser Quantum). Detection was carried out using a Symphony II 1024 × 256 Cryogenic Back-Illuminated Deep-Depletion CCD Detector, also from Horiba Jobin Yvon. The Raman measurements were performed at room temperature, and for nonpolarized measurements, a depolarizer was used to scramble the laser polarization.

### Scanning Electron Microscopy (SEM), Transmission Electron Microscopy with Energy-Dispersive X-Ray (TEM-EDX), Microanalysis, and High-Resolution Transmission Electron Microscopy (HRTEM)

The surface morphology and elemental distribution were analyzed by using SEM equipped with an EDX unit, and data acquisition was performed with a silicon drift detector (Oxford Instruments; UK). The h-WO_3_ powder as well as h-WO_3_@Ti/Ni mesh prepared by the hydrothermal method were washed two times by C_2_H_5_O and H_2_O and then dried at 60 °C for 24 h before investigation. The surface morphology of h-WO_3_@Ni was directly analyzed by SEM on a Ni foam substrate (Figure S5a–d), while the microstructure and crystallinity of h-WO_3_ were investigated by peeling h-WO_3_ from the substrate surface. First, the h-WO_3_@Ni sample was immersed in C_2_H_5_OH and ultrasonicated for 5 min; then, the solution with peeled h-WO_3_ was transferred to the TEM grid for investigation, and the results are displayed in Figure S5e–g.

### Electrochemical Measurement

All electrochemical measurements were conducted using a computer-controlled multichannel potentiostat/galvanostat (VMP300; Biologic; France). The CAPode device is a two-terminal device with WE and CE configurations (Figure 8a). The CAPode device was tested using a standard VMP channel setup (Figure 8b). The G-Cap (Figure 8c) is a three-terminal device with WE, GE, and a shared CE between two internal circuits. The operation of the G-Cap device includes applying a voltage between WE and CE (*U*_WE–CE_) for the internal capacitor, and to switch the G-Cap “on” or “off,” a bias voltage is applied between GE and CE (*U*_GE–CE_). When both systems are operating, two currents are flowing in the system noted as *I*_WE–CE_ and *U*_GE–CE_ for the applied *U*_WE–CE_ and *U*_GE–CE_, respectively. Since under standard conditions it is not possible to monitor two flowing currents at the same time, the CE to GR step is further used as it permits and measures multiple currents flowing at the same time. Therefore, for testing the G-Cap device, two channels of the VMP device were connected under CE to ground setup instead of the standard connection setup. Under this setup, the cables’ functions are different. Figure S8d represents the function of the new cable connection of the CE toground setup, where the CE to ground setup includes using the black cable (GR) and light red cable (REF) as CE cables, the dark blue cable and light blue cable (REF3) as WE cables, the dark red cable as GR, and the white cable as RE cables. Furthermore, the G-Cap configuration with a connection cable of two channels was represented (Figure S8e, f).

### Rectification Ratios

Rectification ratio I is calculated at the cut off voltage (±1 V) based on eq 8 from the cyclic voltammogram (where RR_I_ is the rectification ratio I [−], *I*_OV_ is the current (absolute value) registered at maximum applied voltage in open polarization [A], and *I*_BV_ is the current (absolute value) registered at maximum applied voltage in blocked polarization [A]).

8 $${\mathrm{RR}}_{I}=\frac{I_{\mathrm{OV}}}{I_{\mathrm{BV}}}$$

When the *I–t*-curves are used, RR_I_ is calculated for each measuring point by dividing the absolute current value registered for open and blocked polarization.

Rectification ratio II is calculated based on eq 9 from cyclic voltammogram (where RR_II_ is the rectification ratio II [%], *A*_OV_ is the integral (absolute value) under the current curve registered at open polarization [A V], and *A*_FV_ is the integral (absolute value) under the current curve registered at open and blocked (full) polarization [A V]).

9 $${\mathrm{RR}}_{\mathrm{II}}=\frac{A_{\mathrm{OV}}}{A_{\mathrm{FV}}}$$

The higher the capacity under ″open″ polarization, the higher the RR_II_ ratio (according to eq 9). However, the RR_I_ ratio (eq 8) cannot be related to the capacity because a high current at the maximum voltage under ″open″ polarization does not necessarily mean a high capacity of the entire system, but a redox peak can lead to high current.

### Logic Gates

The optimized device is tested in both simple and complex logic gate configurations using a software and a computerized waveform generator connected to an oscilloscope provided by SweepMe! (Germany). Two devices were connected to form an OR gate circuit and an AND gate circuit (Figure S23), allowing for an evaluation of their basic responses under standard conditions. The power consumed by logic gates is calculated using the power equation *P* = *IU*, where *P* is the power in watts (W), *I* is the current in amperes (A), and *U* is the input voltage in volts (V). In the case of CAPode devices, the power consumption is defined as the energy required to drive the current to the output, calculated as *P* = *IU*_d_, with *U*_d_ representing the voltage drop across the CAPode, whereas the power dissipation, which represents the energy transferred to the next logic gate, is alternatively measured across a resistor that drops the voltage to 0 V, given by *P* = *IU*_out_, where *U*_out_ is the voltage across the resistor. The electronic circuits of the basic logic gates are illustrated in Figure S23. The current flow in each circuit is measured before the dissipation resistor, which also allows for adjusting the circuit’s current. A 4.3 kΩ resistor is used in the configurations of both the AND and OR gates. Furthermore, the voltage across the CAPode devices is monitored by using a potentiostat/galvanostat VMP.

## Supplementary Material



## Data Availability

The raw data used in this study are openly available on Zenodo at 10.5281/zenodo.15387744.

## Notes

List of materials used; synthesis procedure of h-WO_3_-based electrodes; description of the XPS measurements; description of the XRD measurements and corresponding results; description of the Raman spectroscopy measurements and results for the h-WO_3_@Ti sample; description of SEM, TEM-EDX, and HRTEM measurements with corresponding images; electrochemical measurements (including results for systems with different electrolytes, various H_2_SO_4_ concentrations, different WO_3_-based electrodes, varying WE/CE mass ratios, three-electrode configurations, aging tests, and EIS measurements); and logic gate results (PDF)
