# Supplementary material for: Nanostructured h‑WO3‑Based Ionologic Gates with Enhanced Rectification and Transistor Functionality
Source: ACS Nano. 2025 May 26;19(22):20655–71. doi: 10.1021/acsnano.5c02035 (PMC12164519; doi:10.1021/acsnano.5c02035)
Supplement: Supplementary file 1 [file nn5c02035_si_001.pdf]

Supplementary Information

Nanostructured h-WO_3-Based Ionologic Gates with Enhanced Rectification and Transistor Functionality

*Ahmed Bahrawy,[1] Przemyslaw Galek,[1] * Christin Gellrich,[1] Nick Niese,[1] Mohamed A. A. Mohamed,[2,3] Martin Hantusch,[2] Julia Grothe,[1] and Stefan Kaskel[1,4] **

[1] Inorganic Chemistry I, Technische Universität Dresden, Bergstrasse 66, 01069 Dresden, Germany

[2] Leibniz Institute for Solid State and Materials Research Dresden, Helmholtzstraße 20, 01069 Dresden, Germany

[3] Department of Physics, Faculty of Science, Sohag University, 82524 Sohag, Egypt

[4] Fraunhofer Institute for Material and Beam Technology (IWS), Winterbergstraße 28, 01277 Dresden, Germany

Table of contents

1. Synthesis procedure.....2
2. X-ray Powder Diffraction (XRD).....2
3. Raman spectroscopy.....3
4. Scanning Electron Microscopy (SEM), Transmission Electron Microscopy with Energy-Dispersive X-Ray (TEM-EDX), Microanalysis, and High-Resolution Transmission Electron Microscopy (HRTEM).....3
5. Electrochemical measurement.....5
6. Logic gates.....14

1. Synthesis procedure

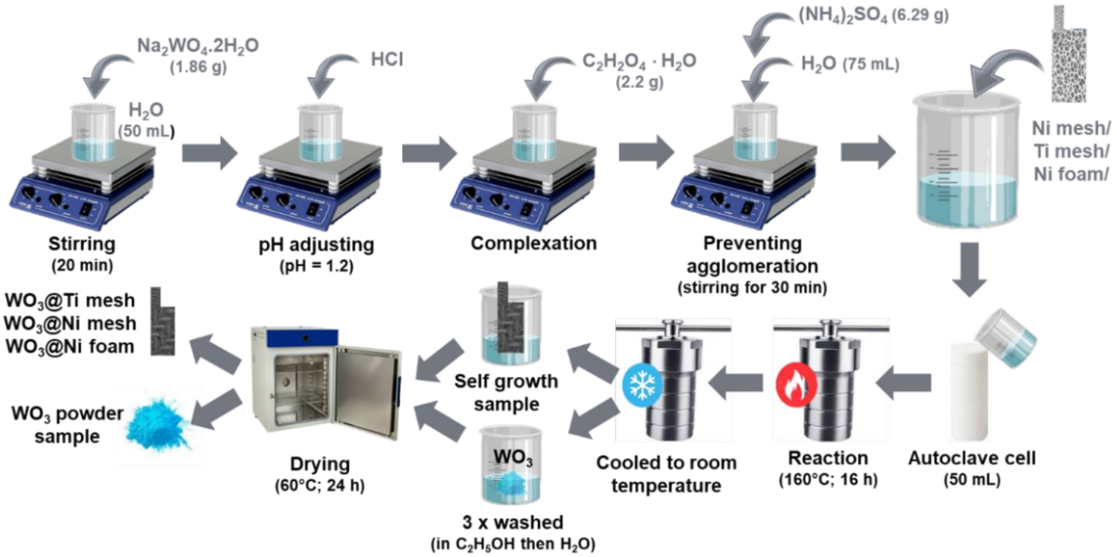

Figure S1. Schematic diagram illustrating the hydrothermal synthesis process of WO_3, and WO_3@Ni, WO_3@Ti electrodes.

2. X-ray Powder Diffraction (XRD)

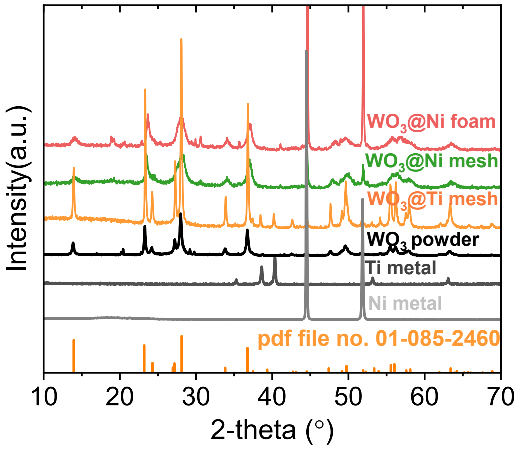

Figure S2. XRD patterns of Ni substrate, h-WO_3 powder, h-WO_3@Ti mesh, h-WO_3@Ni mesh, and h-WO_3@Ni foam substrates.

3. Raman spectroscopy

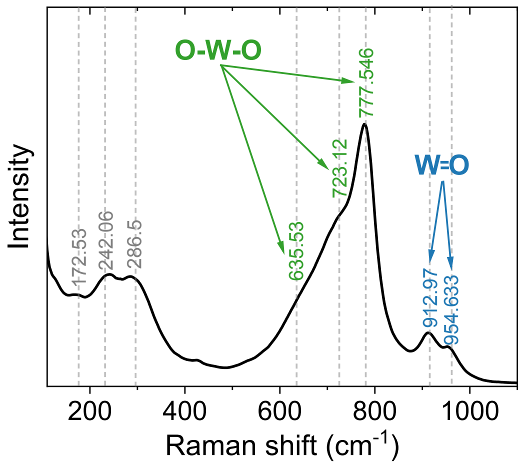

Figure S3. Raman spectrum of the hexagonal h-WO_3@Ti sample prepared by hydrothermal method without treatment.

4. Scanning Electron Microscopy (SEM), Transmission Electron Microscopy with Energy-Dispersive X-Ray (TEM-EDX), Microanalysis, and High-Resolution Transmission Electron Microscopy (HRTEM)

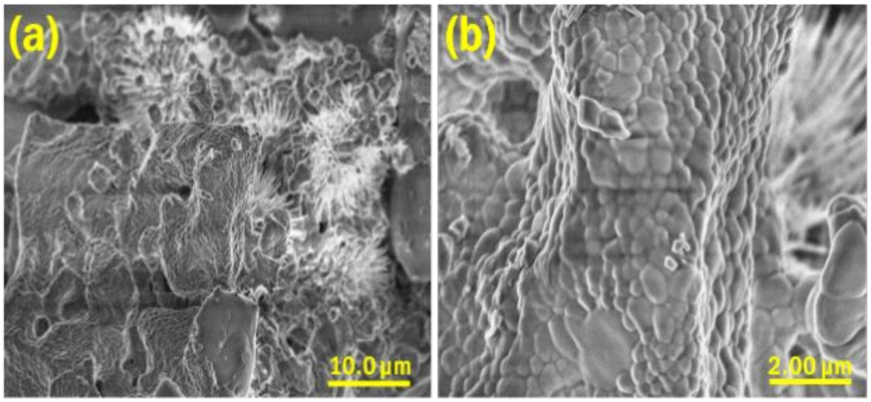

Figure S4. SEM images of as-prepared h-WO_3 powder sample at varying magnification.

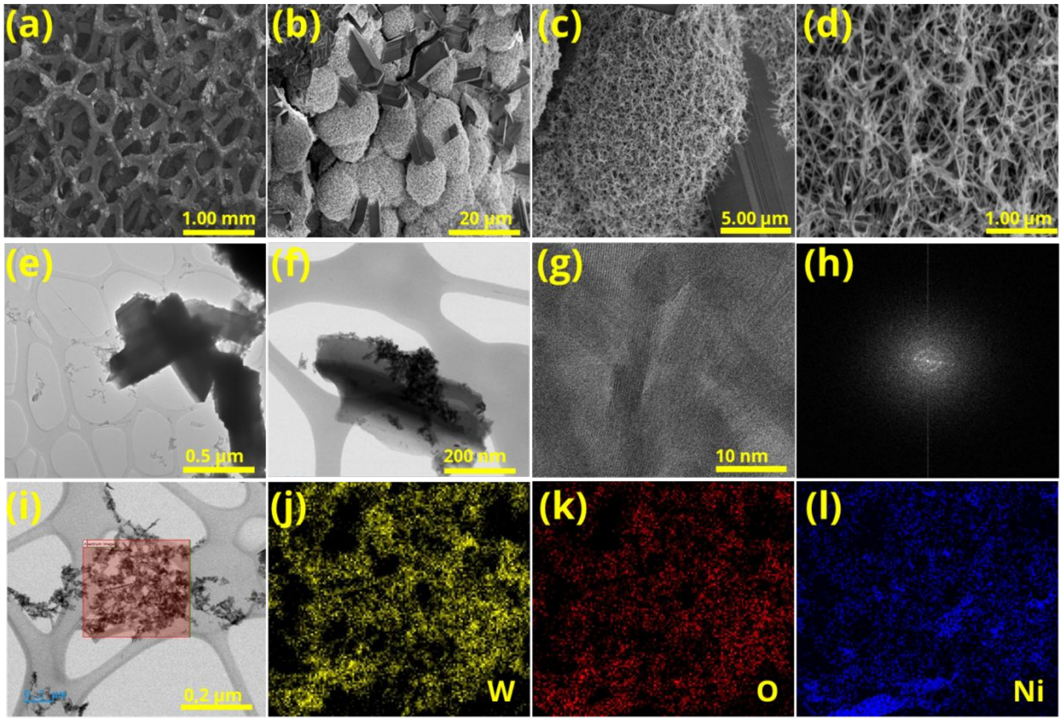

Figure S5. surface characterization of prepared h-WO_3@Ni. (a-d) represent surface morphology at different magnification. (e-g) TEM images of prepared h-WO_3, (h) SAED pattern of the prepared h-WO_3/NiOx, and (i-l) Elemental distribution of W, O, and Ni elements.

The surface composition of h-WO_3 prepared on Ti/Ni mesh and Ni foam was analyzed, with the results presented in **Figure S6**. For each substrate, two EDX analyses are provided, showing variations in elemental concentrations. Notably, the h-WO_3 layer on the Ti substrate exhibited a higher purity compared to the samples on Ni mesh and Ni foam, indicating a more uniform composition with minimal contamination from other elements.

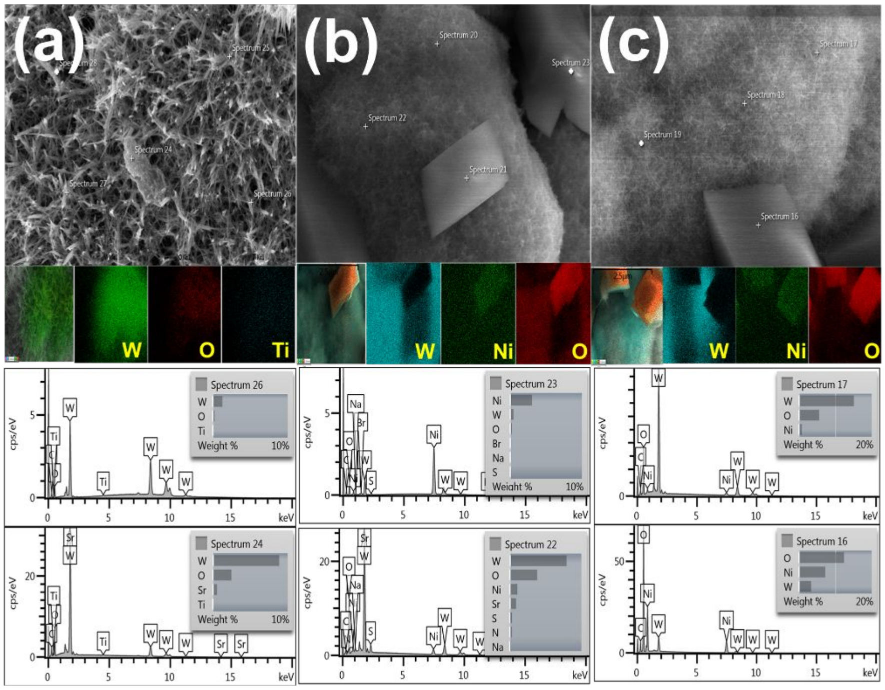

Figure S6. The SEM-EDX and elemental mapping of (a) h-WO_3@Ti, (b) h-WO_3@Ni mesh, and (c) h-WO_3@Ni foam.

5. Electrochemical measurement

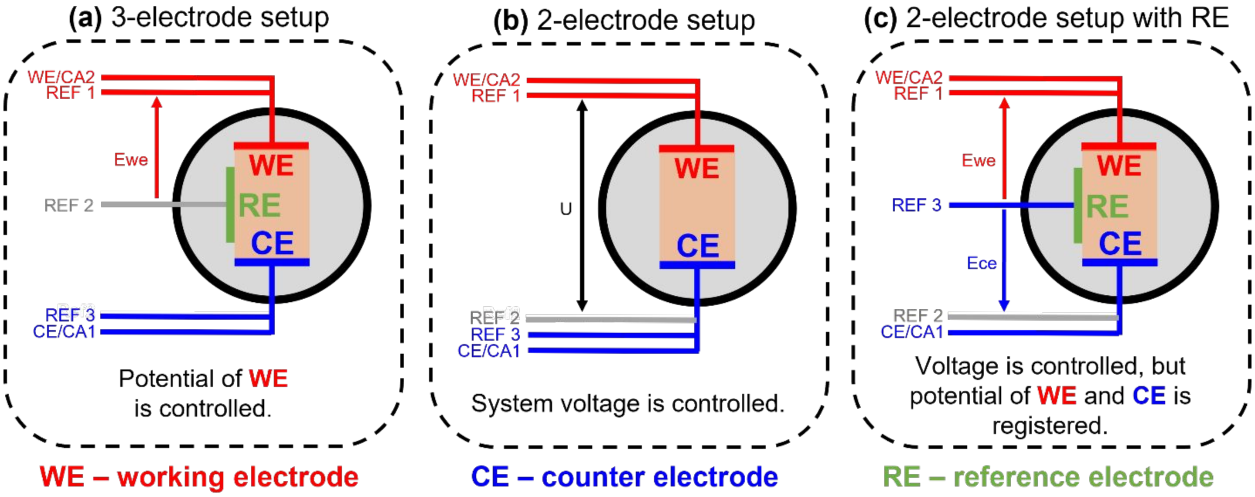

Figure S7. Schematic representation of electrochemical setups used in conducted experiments: (a) 3-, (b) 2-, and (c) 2-electrode setup with RE.

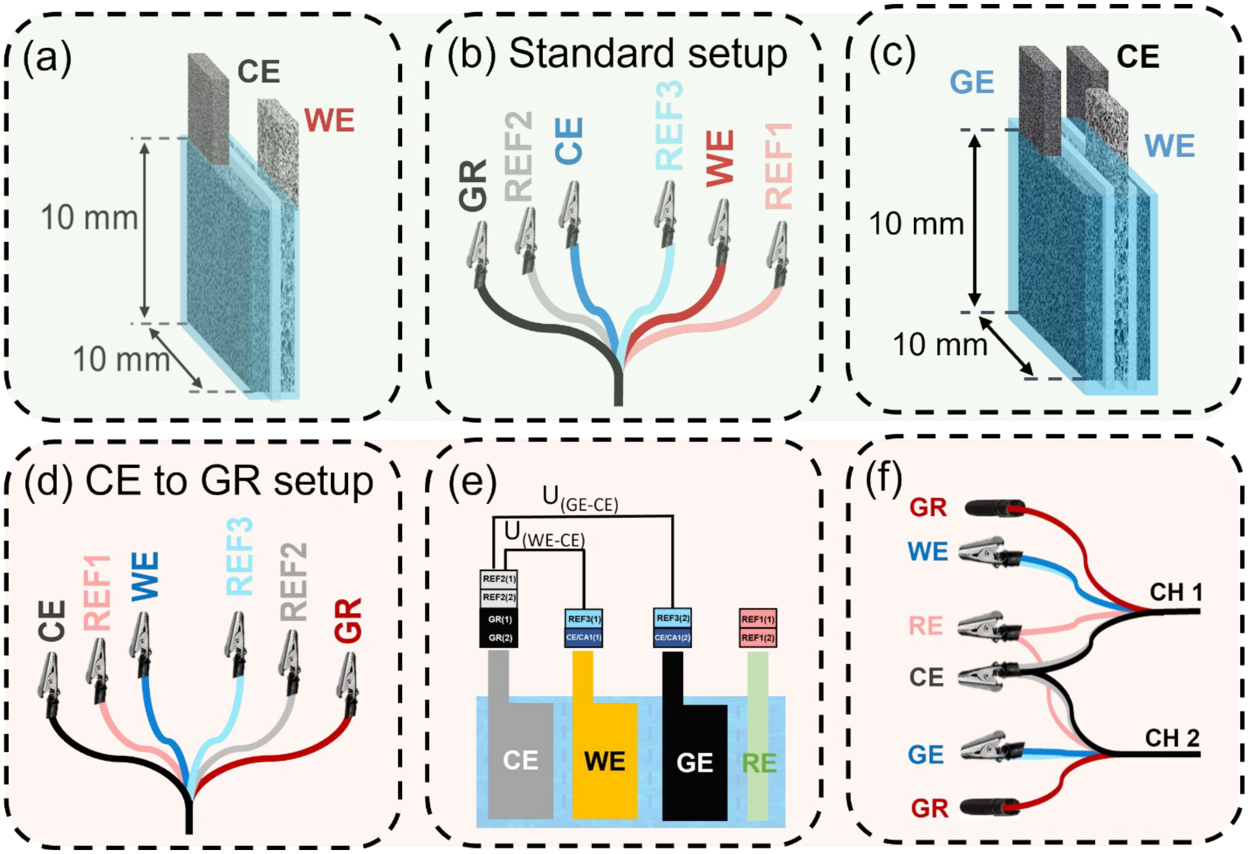

Figure S8. Schematic representation of device configuration and setups used in conducted experiments: (a) CAPode device structure, (b) standard VMP channel setup, (c) G-Cap device structure, (c) VPM CE to GR setup, (e) G-cap configuration with two channels connection and with RE, and (f) model for two connected VPM channels for G-Cap device.

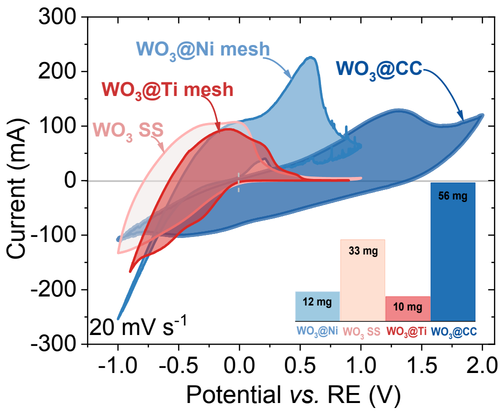

Figure S9. Comparison of CV curves registered for h-WO_3@Ti mesh, h-WO_3@Ni mesh, h-WO_3@CC and h-h-WO_3 free-standing WE electrodes (0.5 mol L^{-1} H_2SO_4 as an electrolyte). The inset shows the corresponding active mass of each electrode.

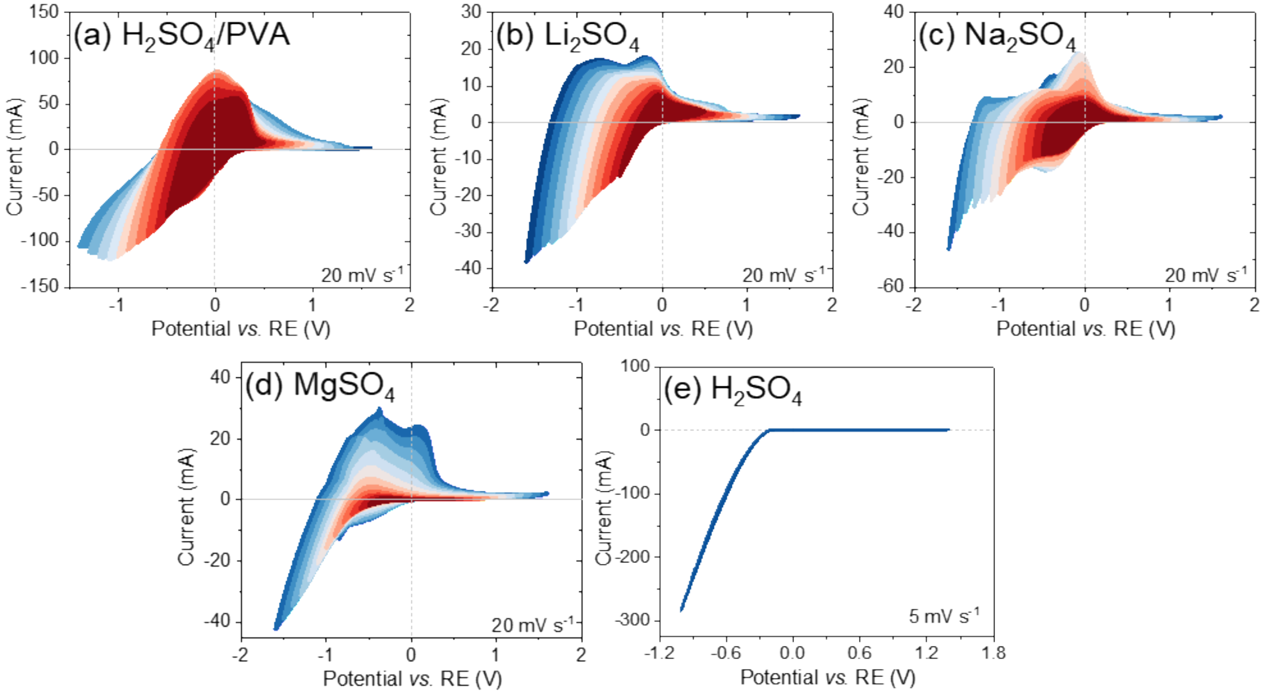

Figure S10. CV curves of h-WO_3@Ti at different potential windows in 0.5 mol L^{-1} (a) H_2SO_4/PVA, (b) Li_2SO_4, (c) Na_2SO_4, (d) $MgSO_4$ as electrolytes with extended potential window across 0 V *vs.* RE, and (e) the CV curve of Ti substrate in 0.5 mol L^{-1} H_2SO_4.

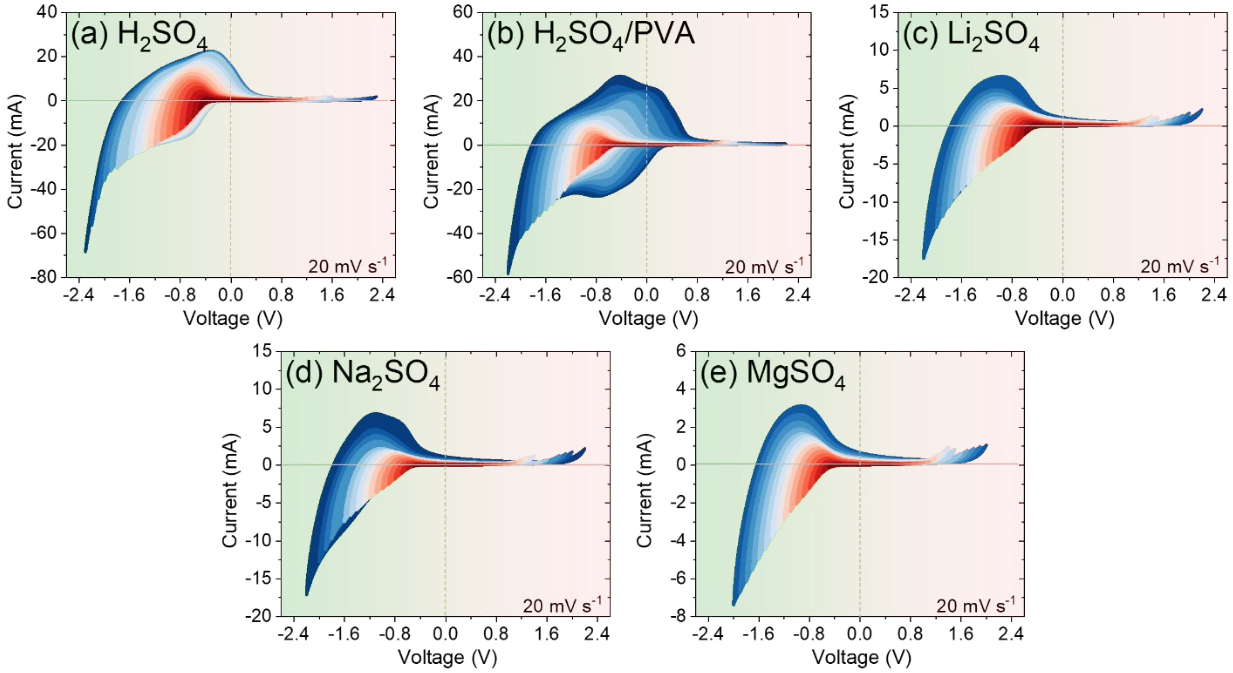

Figure S11. CV curves registered for CAPodes with 0.5 mol L^{-1} (a) H_2SO_4, (b) H_2SO_4/PVA, (c) Li_2SO_4, (d) Na_2SO_4, and (f) $MgSO_4$ as electrolytes at varying voltage window from ±0.5 to ±2.2 V.

The CAPode performance was tested in different electrolytes (**Figure S12b**) and the corresponding RR_I are calculated at different scan rates (**Figure S12b**).

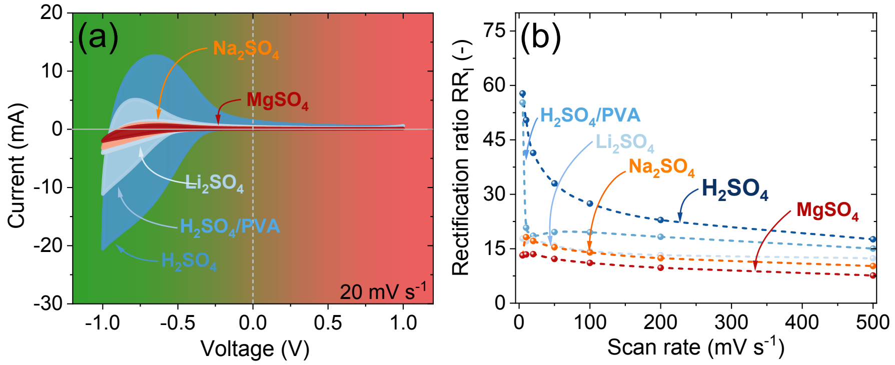

Figure S12. (a) Comparison of CV curves at ±1 V voltage window and (b) the calculated RR_I at different scan rates for CAPodes with different electrolytes.

Table S1. Rectification ratios (RR_I and RR_{II}) of the CAPode systems developed in this study compared with previously reported devices.

CAPode material	Electrolytes	RR_I [-]	RR_{II} [%]	Scan rate [mV s^{-1}]	Mechanism
**$C_{4.80}$ | $TBABF_4$(AN) | $C_{0.60}$**[a)]	1 mol L^{-1} $TBABF_4$ in AN	12	90	20	Ion sieving
**YP-50F/YP-50F (1:10) mass ratio**[b)]	Poly cation ionic liquid ((EMIM)$_n$·PSTFSI)	8.16	74	20	
	Poly anion ionic liquid (PVBIm·(TFSI)$_n$)	8.14	85	20	
**$ZnCo_2O_4$/YP-50F CAPode**[c)]	1 mol L^{-1} KOH	3.4	79	200	Redox
**Molybdenum-oxide-based CAPode**[d*)]	9 mol L^{-1} $NaClO_4$ + $nM$$HClO_4$	136[f)]	95.9	20	Intercalation
**$C_{0.87}$ | 20% EmimPAF (ACN) | $C_{1.5}$**[e)]	20% EmimPAF (ACN)	10	90	5	Ion sieving
**$C_{0.87}$ | 1M $TPABF_4$ (ACN) | $C_{1.5}$**[e)]	1 mol L^{-1} $TPABF_4$ (ACN)	14	80		
**$Ni_3Bi_2S_2$@Ni | 1 mol L^{-1} KOH | Cabot@Ni**[f)]	1 mol L^{-1} KOH	**37**	96	10	Redox
**WOx, MoOx, VOx/ LiPON/ WOx, MoOx, VOx**[g)]	lithium phosphorus oxy-nitride (LiPON)	20	83	5	Redox
**h-WO_3@Ti | 0.5 mol L^{-1} Mg_2SO_4 | Cabot@Ni**[h)]	0.5 mol L^{-1} Mg_2SO_4	13.15	85.5	5	Intercalation
**h-WO_3@Ti | 0.5 mol L^{-1} Na_2SO_4 | Cabot@Ni**[h)]	0.5 mol L^{-1} Na_2SO_4	13.199	83.1	5	Intercalation
**h-WO_3@Ti | 0.5 mol L^{-1} Li_2SO_4 | Cabot@Ni**[h)]	0.5 mol L^{-1} Li_2SO_4	17.77	87.7	5	Intercalation
**h-WO_3@Ti | 0.5 mol L^{-1} PVA/H_2SO_4 | Cabot@Ni**[h)]	0.5 mol L^{-1} PVA/H_2SO_4	55.27	94.5	5	Intercalation
**h-WO_3@Ti | 0.5 mol L^{-1} H_2SO_4 | Cabot@Ni**[h)]	0.5 mol L^{-1} H_2SO_4	57.78	95.8	5	Intercalation

[a)] E. Zhang, N. Fulik, G.-P. Hao, H.-Y. Zhang, K. Kaneko, L. Borchardt, E. Brunner, S. Kaskel, Angewandte Chemie International Edition 2019, 58, 13060.; [b)] J. Feng, Y. Wang, Y. Xu, H. Ma, G. Wang, P. Ma, Y. Tang, X. Yan, Advanced Materials 2021, 33, 2100887.; [c)] P. Tang, W. Tan, F. Li, S. Xue, Y. Ma, P. Jing, Y. Liu, J. Zhu, X. Yan, Advanced Materials 2023, 35, 2209186.; [d)] H. Ma, J. Liang, J. Qiu, L. Jiang, L. Ma, H. Sheng, M. Shao, Q. Wang, F. Li, Y. Fu, J. Wang, E. Xie, Y. Chai, W. Lan, Advanced Materials 2023, 35, 2301218.; [e)] H. Zhou, P. Li, E. Zhang, J. Kunigkeit, X. Zhou, K. Haase, M. Rita Ortega Vega, S. Wang, X. Xu, J. Grothe, S. C. B. Mannsfeld, E. Brunner, K. Kaneko, S. Kaskel, Angewandte Chemie International Edition n/a, e202305397; f) Bahrawy, A., Galek, P., ification ratio calculate by Gellrich, C., Grothe, J. & Kaskel, S. Advanced Functional Materials n/a, 2405640. [g)] Wang, D.; Yang, B.; Zhou, Z.; Zhang, Z.; Wu, Z.; Huang, X.,. Angewandte Chemie (International ed. in English) 2025, 64 (12), e202421913. [h)] this work. * Rectification ratio calculated by second method.

The mass ratio between WE, and CE was optimized (**Figure S13**). 10 mg of h-WO_3 was hydrothermally grown on the Ti surface and employed as WE. On the other hand, different masses of NC coated on the Ti surface were used to balance the charge of the intercalation reaction on WE and employed as a CE.

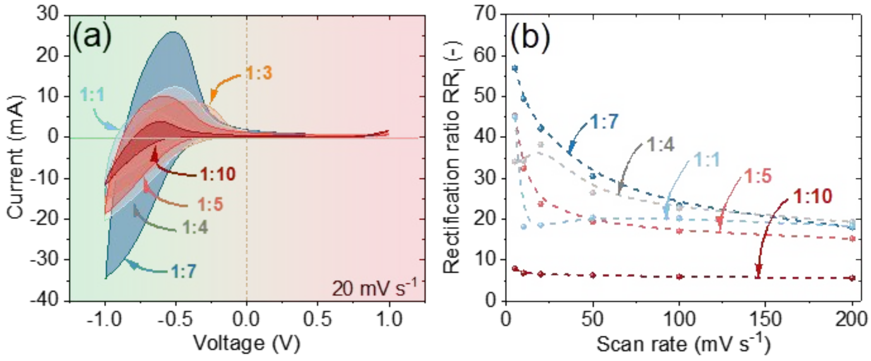

Figure S13. (a) CV curves of the constructed h-WO_3@Ti│0.5 mol L^{-1} H_2SO_4│AC@Ti CAPode with different WE/CE mass ratio and (b) the calculated RR_I for systems at different scan rates.

Generally, the charge generated by redox reaction or intercalation reaction is much higher than that accumulated in EDL, therefore 10, 40, 50, 70, and 100 mg of NC were tested to reach the maximum capacity of the device under open polarization. **Figure S13** represents a comparison between different systems response with mass ratios between WE/CE. The device with a 1:7 mass ratio shows the highest rectification over different scan rates (**Figure S13b**).

The electrolyte concentration plays an important role in the current response, especially under open polarization. Three different concentrations (0.1, 0.5, and 5.0 mol L^{-1}) of sulfuric acid were tested, and the CV results show that the 5 mol L^{-1} sulfuric acid concentration revealed the highest current response and specific capacity compared to other concentrations (**Figure S14**).

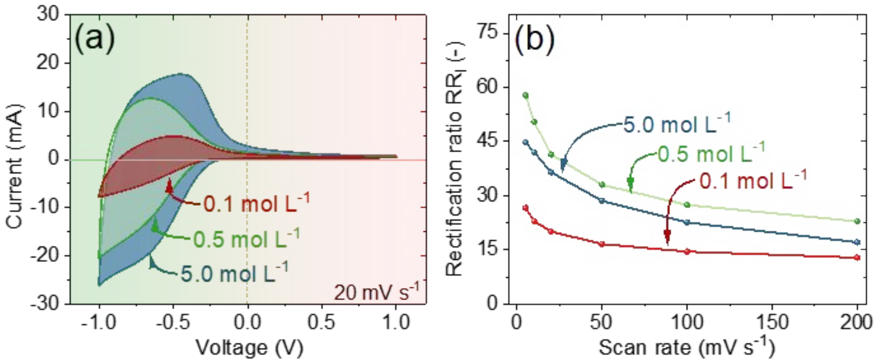

Figure S14. (a) CV curves of the constructed h-WO_3@Ti│X mol L^{-1} H_2SO_4│AC@Ti CAPode with different electrolyte concentration and (b) the calculated RR_I for the system at different scan rates.

However, the device with a 5 mol L^{-1} electrolyte concentration shows the highest current under open polarization as depicted in **Figure S14a** and the device with a 0.5 mol L^{-1} electrolyte showed the highest RR_I at all scan rates **Figure S14b**. Because the CAPode application mainly depends on the ability of the device to rectify the current response, 0.5 mol L^{-1} is selected to be used for further application.

The working mechanism of the proposed device was investigated using in situ electrochemical measurements, employing Galvanostatic Charge-Discharge (GCD) and Cyclic Voltammetry (CV) techniques. The configuration for connecting the device under these measurement conditions is detailed elsewhere.[2] In this setup, the potential distribution across the WE and CE was monitored during the charging and discharging cycles using the GCD technique.

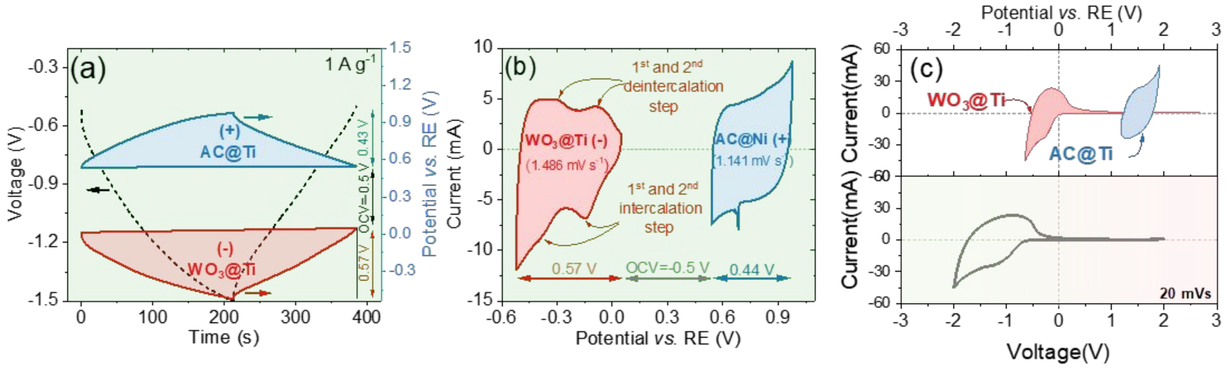

Figure S15. (a) GCD profile (black dashed line) of the system in the range of OCV ↔ -1.5 V, with simultaneous monitoring of the electrochemical potential distribution of the WE and CE (red and blue lines, respectively), (b) corresponding CV curves of WE and CE at scan rates calculated from GCD profiles, and (c) the CV curve of the CAPode (2-electrode setup) in symmetrical window ±2 V (lower part of the plot) and corresponding potential distributions between WE and CE (upper part of the plot).

At -1 V, a single intercalation peak was observed (**Figure 4e**), while applying -1.5 V resulted in two distinct peaks visible in h-WO_3@Ti(-) electrode (red curve in **Figure S15b**). These findings are consistent with the results from 3-electrode setup (**Figure S9**; h-WO_3@Ti mesh), indicating the device's suitability for extending the voltage window. The current peak visible in the blue CV curve in **Figure S15b** registered for NC@Ti(+) (0.45 V *vs.* RE) is an artifact and should not be interpreted as a redox peak.

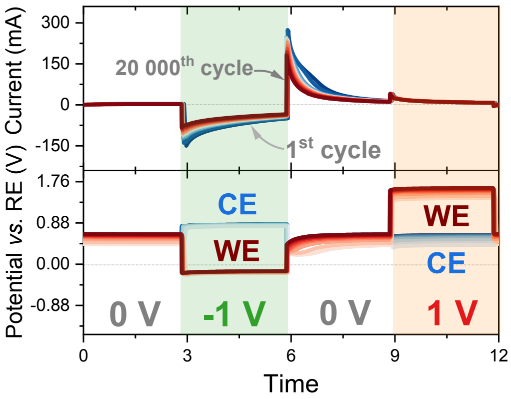

Figure S16. The *I-t* profile of the system under ±1 V (for 3 s each, with a rest step) for 20,000 cycles, combined with potential distribution between WE and CE.

The chargeability of the proposed device was evaluated under different conditions, as shown in **Figure S17**, which presents the current-time (*I-t*) responses over 300 s for two distinct testing sequences.

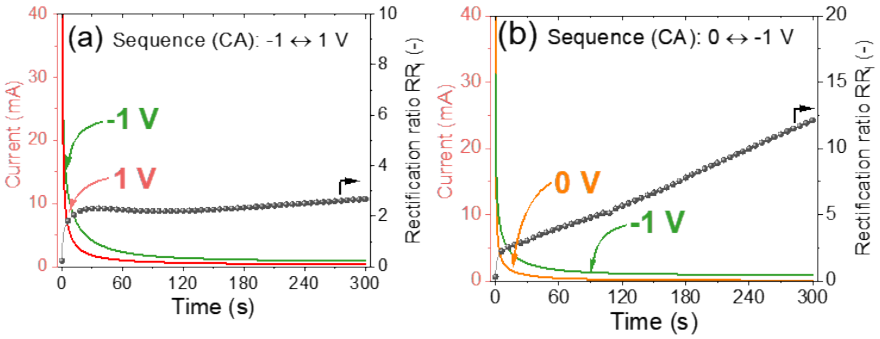

Figures S17. The *I-t* and the rectification response of CAPode for 300 s under (a) ±1 V and (b) under -1/0 V without resting steps.

In the first sequence (**Figure S17a**), the device was directly repolarized from "negative" to "positive" voltage without a rest step, and the corresponding current ratio (R_I) was tracked step-by-step throughout the 300 s duration. In the second sequence (**Figure S17b**), the device was initially polarized to "negative" voltage before proceeding to 0 V, with the current ratio monitored simultaneously. These experiments provide insights into how different repolarization approaches affect the device's chargeability and overall performance.

Long term cyclic stability and capacity/rectification retention are two important factors controlling the overall device response. Therefore, the CAPode device was tested under ±1 V sequence for 20,000 cycles and the current rectification as well as rectification retention were calculated and presented in **Figure S17**. These calculations help assessing the device's ability to maintain consistent rectification performance throughout prolonged cycling.

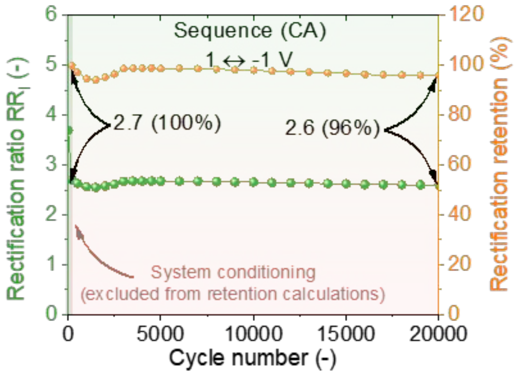

Figure S18. Calculated RR_I and rectification retention during CA measurements over 20,000 cycles. The surface morphology, elemental composition, and elemental mapping of the h-WO_3@Ti electrode was tested after 20,000 cycles to study the electrochemical stability of grown layer (**Figure S18**).

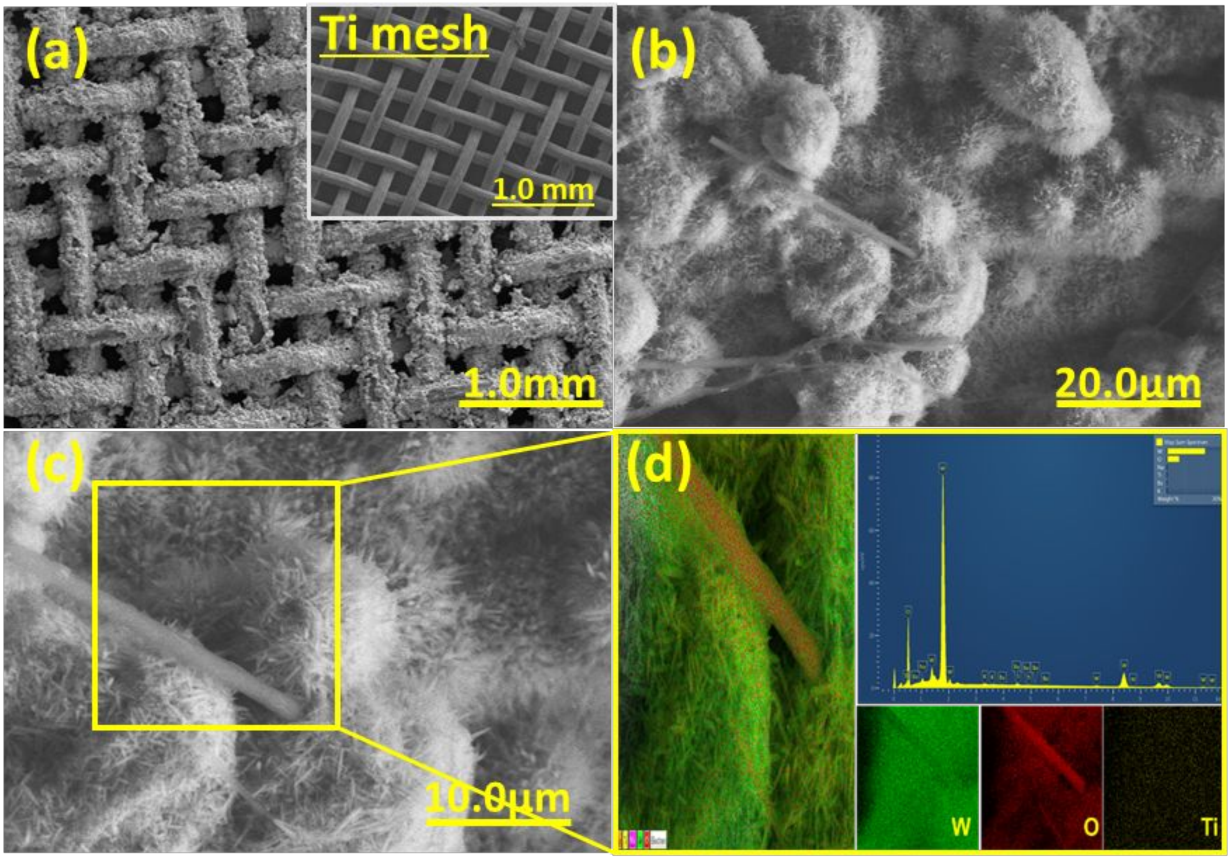

Figure S19. (a-c) SEM (in set Ti mesh), and (d) EDX analysis of h-WO_3@Ti electrode after 20,000 cycles of CAPode repolarization (±1 V).

The crystallinity of h-WO_3@Ti electrode was tested after 20,000 cycle to study the electrochemical stability of grown layer (**Figure S19**).

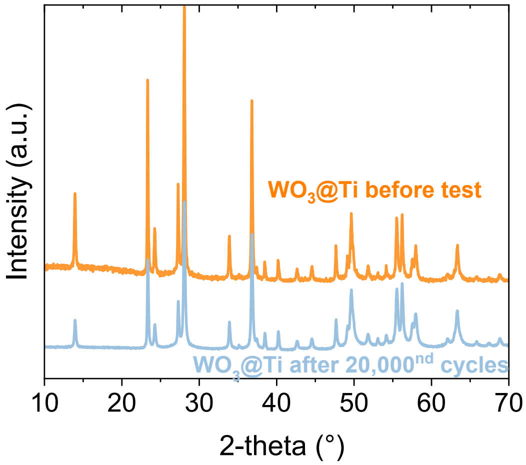

Figure S20. XRD patterns of h-WO_3@Ti electrode before and after 20,000 CA cycles of CAPode repolarization (±1 V).

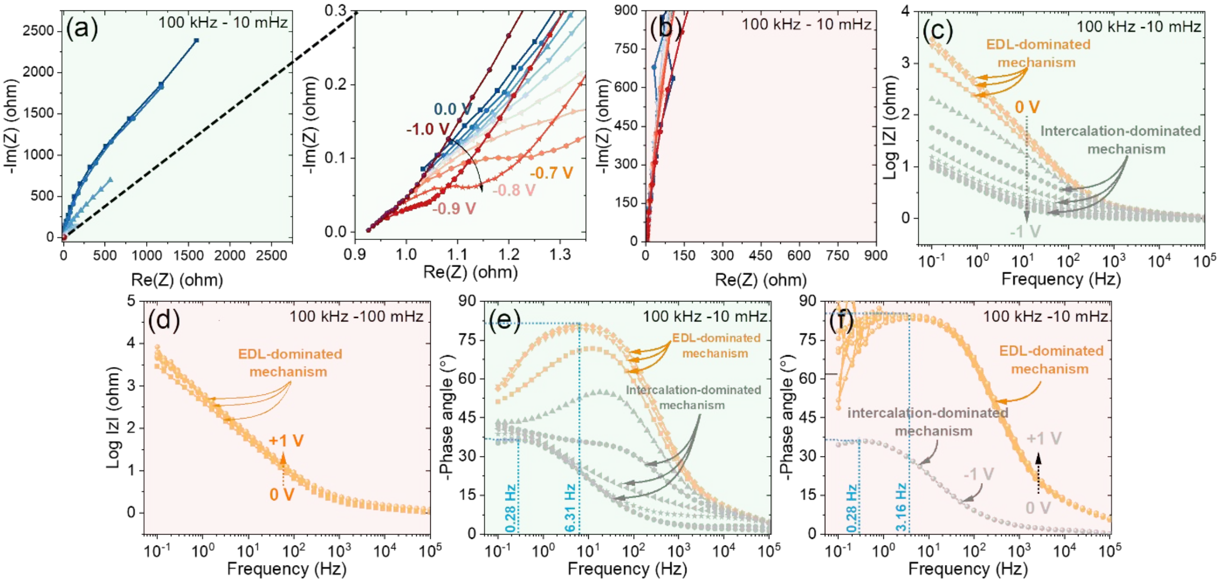

Figurer S21. Nyquist plots of WO_3@Ti│0.5 mol L^{-1} H_2SO_4│AC@Ti CAPode under gradual polarization of (a) 0 → -1 V, (b) 0 → 1 V, bode plots (c) 0 → -1 V, (d) 0 → 1 V, and phase shift plots (e) 0 → -1 V, (f) 0 → 1 V (1 mol L^{-1} H_2SO_4 as the electrolyte).

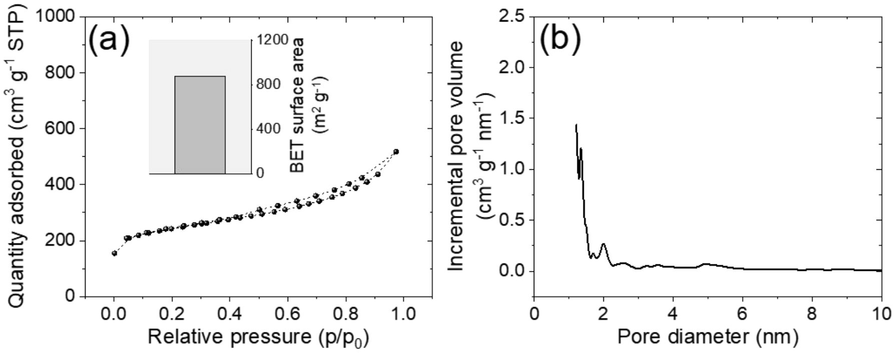

Figure S22. (a) Nitrogen adsorption/desorption isotherm at 77 K for the activated carbon (Cabot) used for CE with materials BET surface area, and (b) the pore size distribution.

6. Logic gates

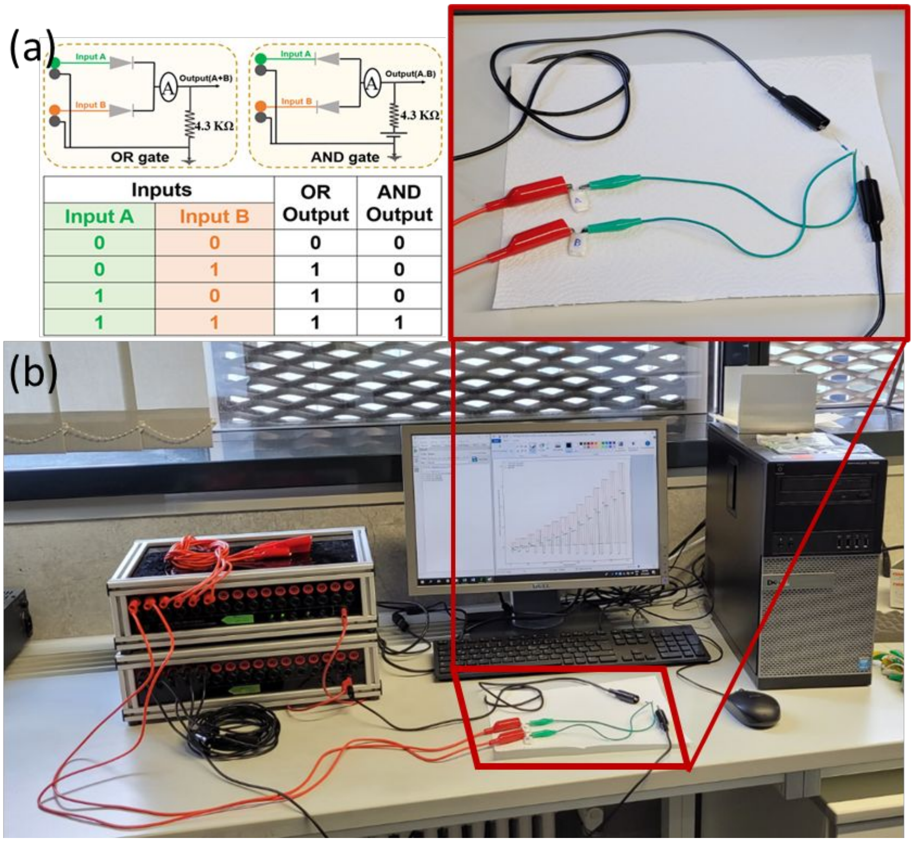

Inputs		OR Output	AND Output
Input A	Input B		
0	0	0	0
0	1	1	0
1	0	1	0
1	1	1	1

Figure S23. The electronic configuration and truth tables for the OR and AND logic gates. (b) A photo shows the CAPodes connected to the computer-controlled generator and oscilloscope.

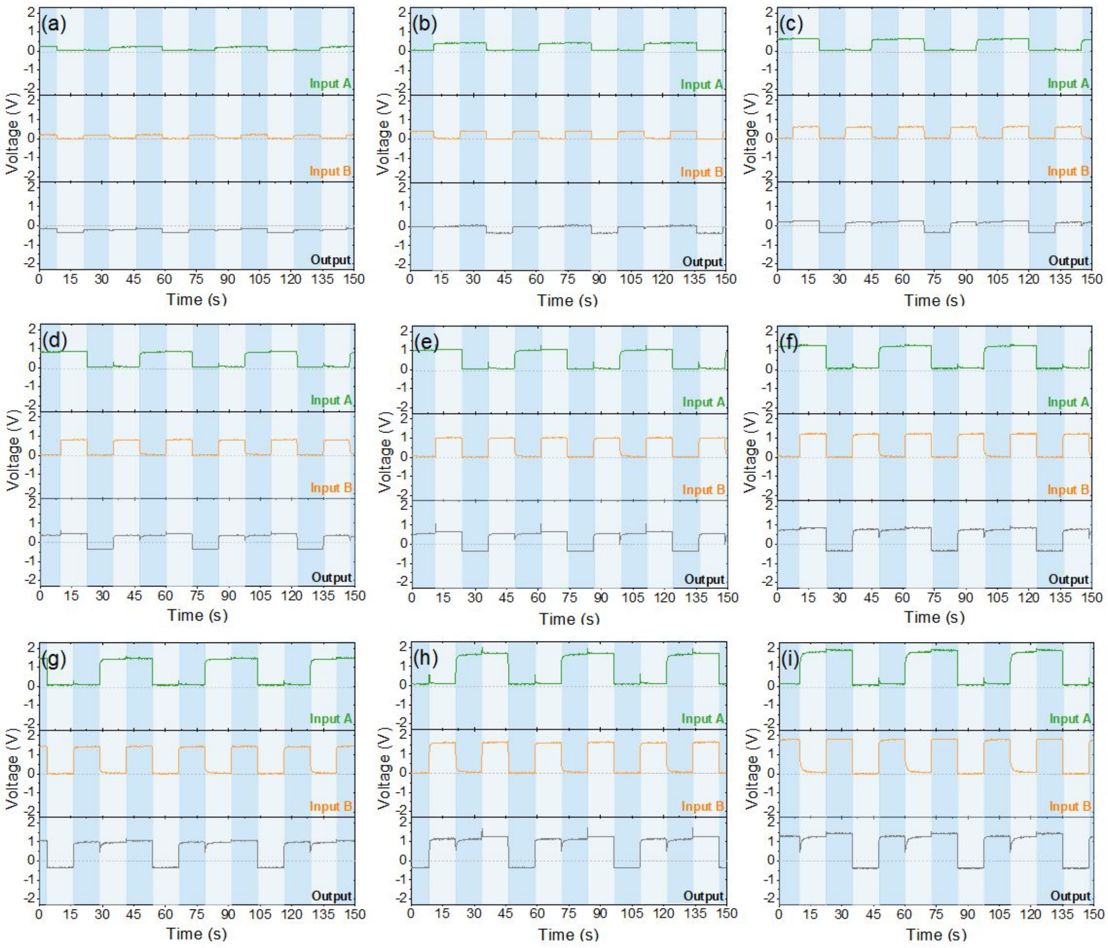

Figure S24. The OR gate output of the constructed CAPodes at applied voltages: (a) 0.2 V, (b) 0.4 V, (c) 0.6 V, (d) 0.8 V, (e) 1.2 V, (f) 1.4 V, (g) 1.6 V, (h) 1.8 V, and (i) 2 V. Input signals A and B operating at frequencies of 50 mHz and 25 mHz, respectively.

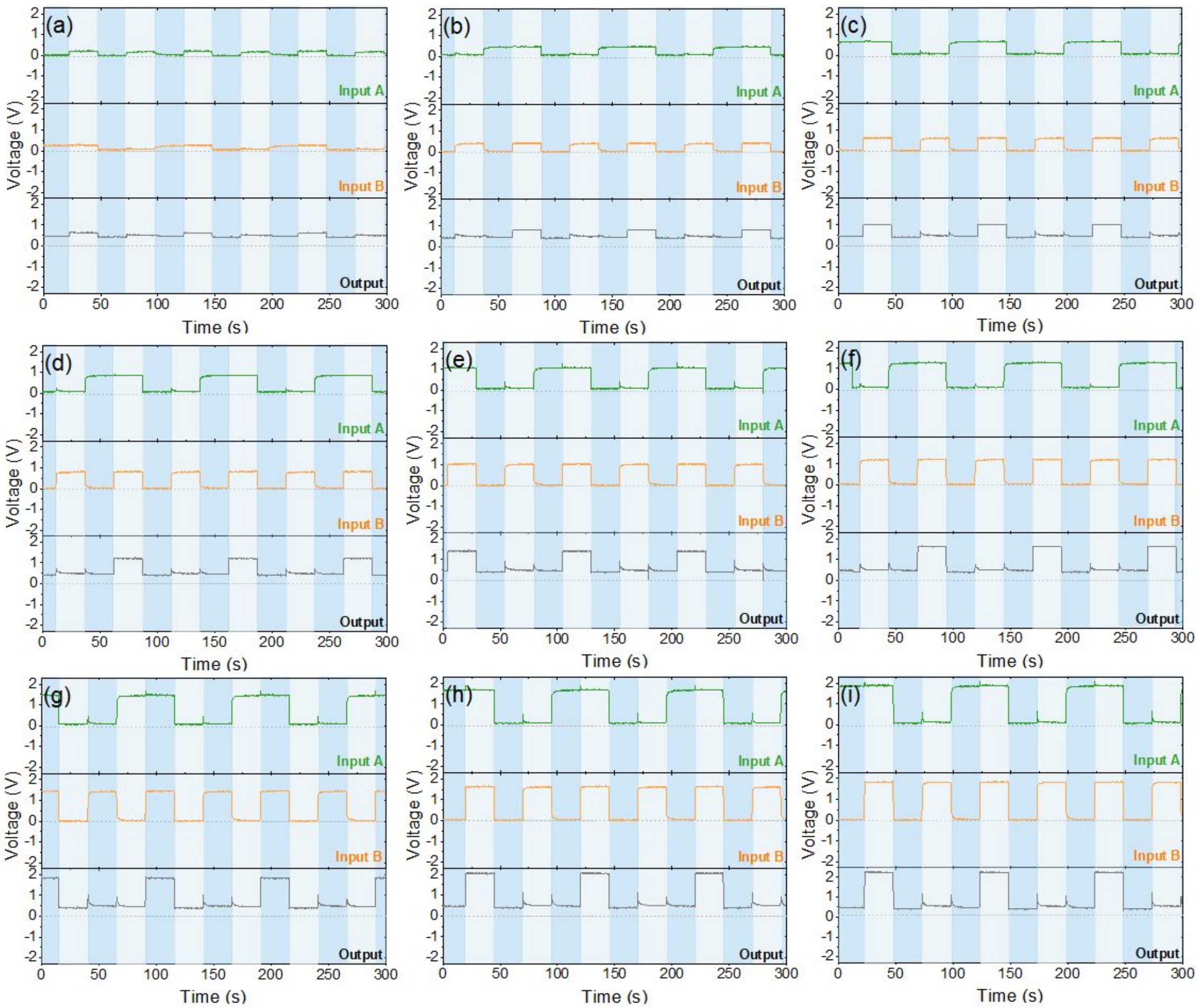

Figure S25. The AND gate results of the constructed CAPodes under applied voltages of: (a) 0.2, (b) 0.4, (c) 0.6, (d) 0.8, (e) 1.2, (f) 1.4, (g) 1.6, (h) 1.8, and (i) 2 V. Input signals A and B operating at frequencies of 25 mHz and 50 mHz, respectively.

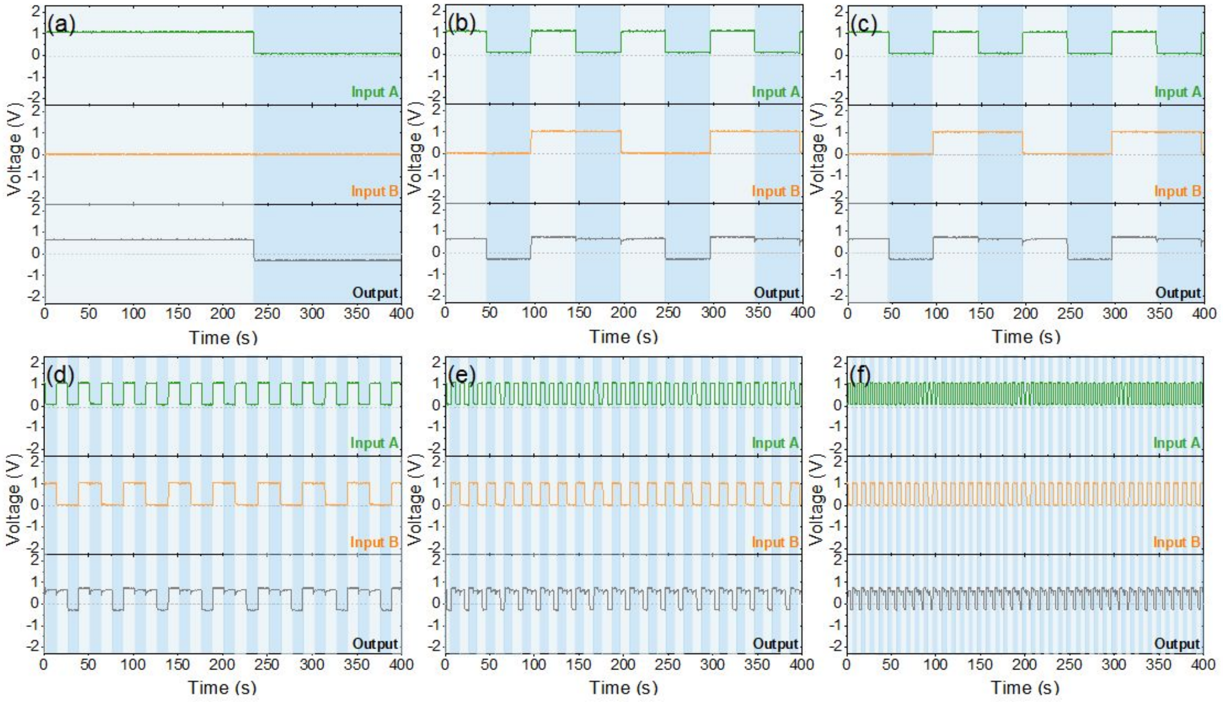

Figure S26. The OR gate results of the constructed devices under applied frequencies of: (a) 2/1, (b) 10/5, (c) 20/10, (d) 40/10, (e) 100/50, and (f) 200/100 mHz, for inputs A and B.

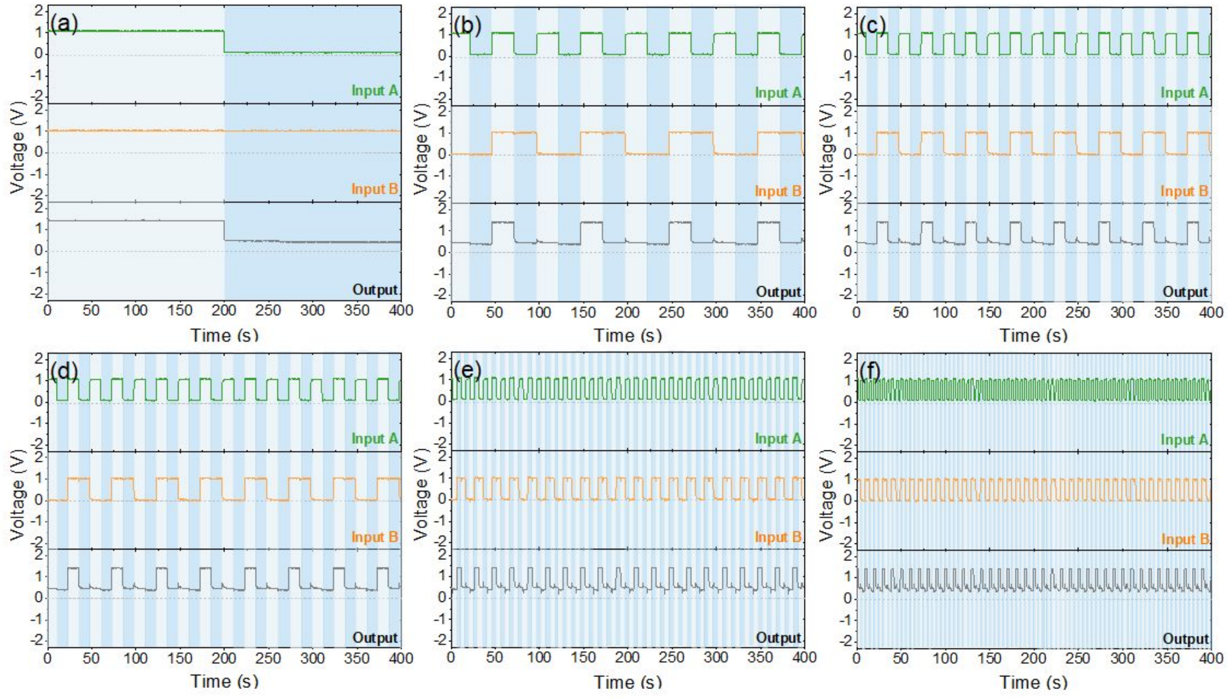

Figure S27. AND gate results of the constructed devices under applied frequencies of: (a) 2/1 mHz, (b) 10/5 mHz, (c) 20/10 mHz, (d) 40/10 mHz, (e) 100/50 mHz, and (f) 200/100 mHz, for inputs A and B.

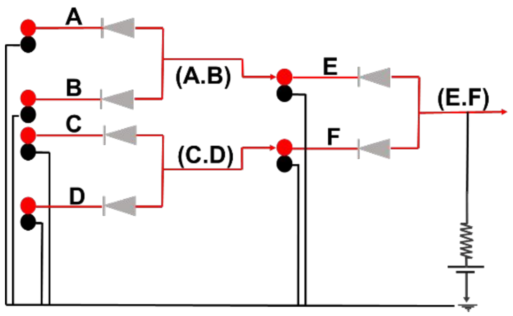

Figure S28. Schematic representation of the electronic connections for the AND/AND/AND configuration.

Table S2. Input sequences (Figure 9 e, c, e, and h) for a system of three interconnected logic gates when the input frequency of A, B, C and D are 0.05, 025, 0.05 and 0.025 Hz).

		**Input sequence**															
Input signals	A	1	0	1	0	1	0	1	0	1	0	1	0	1	0	1	0
	B	1	1	0	0	1	1	0	0	1	1	0	0	1	1	0	0
	C	1	0	1	0	1	0	1	0	1	0	1	0	1	0	1	0
	D	1	1	0	0	1	1	0	0	1	1	0	0	1	1	0	0
Configurations	OR-OR-OR	1	1	1	0	1	1	1	0	1	1	1	0	1	1	1	0
	AND-AND-AND	1	0	0	0	1	0	0	0	1	0	0	0	1	0	0	0
	OR-OR-AND	1	1	1	0	1	1	1	0	1	1	1	0	1	1	1	0
	AND-AND-OR	1	0	0	0	1	0	0	0	1	0	0	0	1	0	0	0

Table S3. Input sequences (Figure 9f and i) for a system of three interconnected logic gates when the input frequency of A, B, C and D are 0.05, 0.0167, 0.05 and 0.0167 Hz).

		**Input sequence**															
Input signals	A	0	1	0	1	0	1	0	1	0	1	0	1	0	1	0	1
	B	1	1	1	0	0	0	1	1	1	0	0	0	1	1	1	0
	C	0	1	0	1	0	1	0	1	0	1	0	1	0	1	0	1
	D	1	1	1	0	0	0	1	1	1	0	0	0	1	1	1	0
Configurations	OR-OR-OR	1	1	1	1	0	1	1	1	1	1	0	1	1	1	1	1
	AND-AND-AND	0	1	0	0	0	0	0	1	0	0	0	0	0	1	0	0
	OR-OR-AND	1	1	1	1	0	1	1	1	1	1	0	1	1	1	1	1
	AND-AND-OR	0	1	0	0	0	0	0	1	0	0	0	0	0	1	0	0

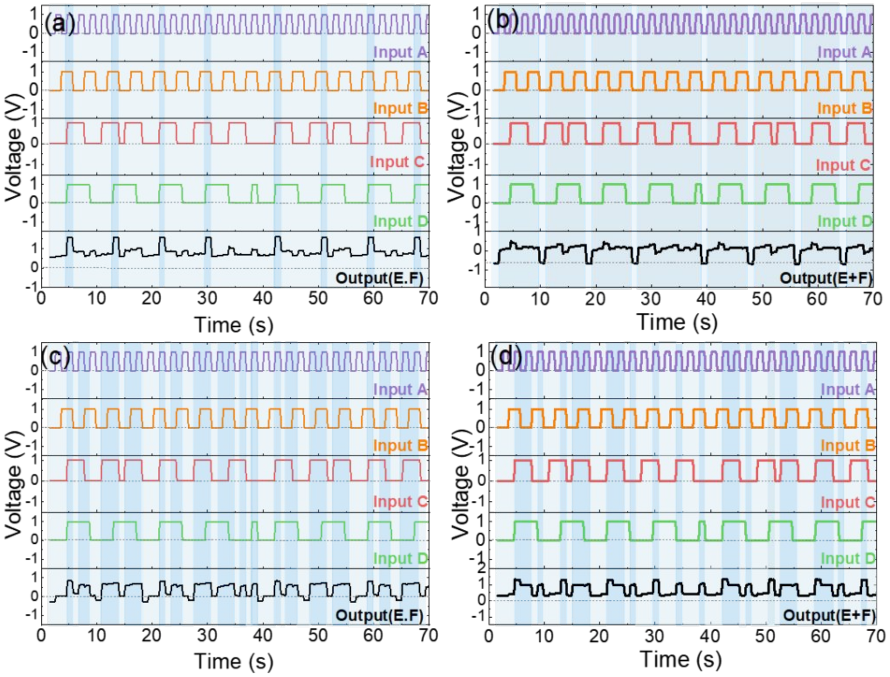

Figure 29. Logic gate results for a complex circuit using three gates with when the input frequency of A B, C and D are 0.5, 0.25, 0.167 and 0.125 Hz: (a) input and output signals observed under the AND/AND/AND configuration, (d) input and output signals observed under the OR/OR/OR configuration, (e) Input and output signals for the OR/OR/AND configuration; and (d) input and output signals observed under the AND/AND/OR configuration,

Table S4. Input sequences for a system of three interconnected logic gates when the input frequency of A B, C and D are 0.5, 0.25, 0.167 and 0.125 Hz).

			**Input sequence**															
Inputs	Gate1	A	0	1	0	1	0	1	0	1	0	1	0	1	0	1	0	1
		B	0	0	1	1	0	0	1	1	0	0	1	1	0	0	1	1
	Gate2	C	0	0	0	1	1	1	0	0	0	1	1	1	0	0	0	1
		D	0	0	0	0	1	1	1	1	0	0	0	0	1	1	1	1
Configurations	OR-OR-OR		0	1	1	1	1	1	1	1	0	1	1	1	1	1	1	1
	AND-AND-AND		0	0	0	0	0	0	0	0	0	0	0	0	0	0	0	1
	OR-OR-AND		0	1	1	1	1	1	1	1	0	1	1	1	1	1	1	1
	AND-AND-OR		0	0	0	1	1	1	0	1	0	0	0	1	0	0	0	1

Figure S30. Signal outputs when both inputs are set to 0 V for the OR and AND gates. Notably, a positive shift occurs in the AND gate output, while a negative shift is observed in the OR gate output, both of which have similar magnitudes. This shift is likely due to the internal resistance of the electrochemical device, although it does not affect the output signal amplitude.[2,3]

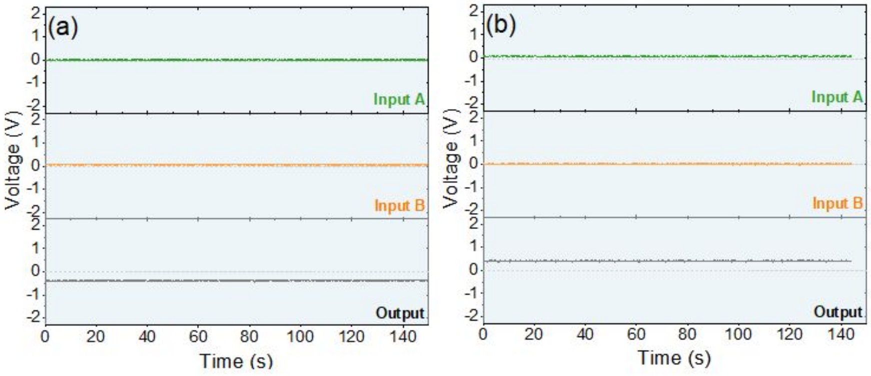

Figure S30. CMOS logic gate results of the constructed devices for (a) OR gate and (b) AND gate when inputs A and B are set to 0 V.

REFERENCES

1 Kalpakli, A. O., Ilhan, S., Kahruman, C. & Yusufoglu, I. Dissolution behavior of calcium tungstate in oxalic acid solutions. *Hydrometallurgy* **121-124**, 7-15, doi: https://doi.org/10.1016/j.hydromet.2012.04.014 (2012).

2 Bahrawy, A., Galek, P., Gellrich, C., Grothe, J. & Kaskel, S. Advanced Redox Electrochemical Capacitor Diode (CAPode) Based on Parkerite (Ni3Bi2S2) with High Rectification Ratio for Iontronic Applications. *Advanced Functional Materials* **n/a**, 2405640, doi: https://doi.org/10.1002/adfm.202405640 (2024).

3 Zhou, H. *et al.* General Design Concepts for CAPodes as Ionologic Devices. *Angewandte Chemie International Edition* **62**, e202305397, doi: https://doi.org/10.1002/anie.202305397 (2023).
